# Mate choice copying in non-human animals: an update of two meta-analyses

**DOI:** 10.1242/bio.062878

**Published:** 2026-10-20

**Authors:** Eduardo S. A. Santos, Aleksandra Milenović, Aneta Arct, Anna Lenz, Ayumi Mizuno, Cassidy Schneider, Christine Sosiak, Erick Lundgren, Hao Qin, Iwo Gross, Jimuel Celeste, Kyle Morrison, Mahi Zakir, Marija Purgar, Santiago Ortega, Sergio Hernandez, Shinichi Nakagawa, Malgorzata Lagisz

**Affiliations:** ^1^ Department of Biological Sciences, Faculty of Science, Collaboration for Open Science and Synthesis in Ecology and Evolution (COSSEE), University of Alberta, Edmonton, AB T6G 2E9, Canada; ^2^ Jagiellonian University, Faculty of Biology, Institute of Environmental Sciences, Gronostajowa 7, 30-387 Kraków, Poland

**Keywords:** Meta-analysis, Mate choice copying, Non-independent mate choice, Social learning, Sexual selection, Social information

## Abstract

Mate choice copying, the use of social information from others' mating decisions, is a taxonomically widespread form of social learning that can shape sexual selection. Two meta-analyses concluded that social information has a moderate positive effect on mate choice; however, both are based on literature searches that are now at least 7 years old. We asked whether the overall average effect they reported remains supported. Using a multilingual, grey-literature-inclusive search across seven languages, we added 69 effect sizes from 29 studies and harmonised the two source metrics, giving 227 Hedges' *g* effect sizes from 87 studies and 172 log odds ratios from 69 studies across 33 species. Reanalysed with phylogenetically informed multilevel models, the overall effect remained positive and significant but was appreciably smaller [Hedges' *g*=0.45, 95% confidence interval (CI): 0.25 to 0.66, versus 0.58; odds ratio=1.81, 95% CI: 1.34 to 2.45, versus 2.71]. Heterogeneity was high, located within studies and among species rather than across the phylogeny. Small-study effects were strong on both scales, and correcting for them drew the mean towards zero. In some species, individuals do copy conspecifics' mate choices, but the effect is smaller, more variable, and more sensitive to publication bias than previously estimated.

## Teaser

**Summary:** Updating two meta-analyses with seven more years of evidence confirms that animals copy each other's mate choices, but the average effect is smaller and less certain than previously reported.

## INTRODUCTION

The process of mate choice allows individuals to evaluate and eventually mate with partners who provide benefits that translate into greater fitness (Rosenthal, 2017). From a social perspective, mate choice events can incidentally convey information that is generally useful about the quality of a mate (Vakirtzis, 2011). When individuals observe these events, acquire social information, and use it to inform their own mating decisions, we call this process mate choice copying (Wade and Pruett-Jones, 1990). As a form of social learning, mate choice copying is theorized to evolve when the costs of independently sampling mates are high, information is limited, or the quality of a potential mate is difficult to assess (Wade and Pruett-Jones, 1990; Vakirtzis, 2011). By using social information from conspecifics, individuals can reduce decision times and mitigate the energetic costs or predation risks of assessing competing choices (Valone and Templeton, 2002).

Mate choice copying is taxonomically widespread and has been experimentally documented across a wide range of non-human animals, including mammals, birds, fish, and arthropods (Vakirtzis, 2011; Jones and DuVal, 2019; Davies et al., 2020). It is important to understand the mechanisms and occurrence of mate choice copying because socially mediated mating decisions can influence the strength and dynamics of sexual selection within a population (Verzijden et al., 2012). Furthermore, when observers form a generalised preference for specific phenotypes rather than particular individuals, this behaviour can facilitate the widespread cultural transmission of mating preferences and ultimately modify the course of evolutionary change (Pruett-Jones, 1992; Varela et al., 2018). Two independent meta-analyses, by Jones and DuVal (2019) and Davies et al. (2020), have examined the magnitude, moderators, and evolutionary implications of mate choice copying using partly different scopes and analytical approaches.

In general, these two meta-analyses (Jones and DuVal, 2019; Davies et al., 2020) found that the presence of social information was moderately and positively associated with an observer's likelihood of choosing that potential mate, compared with the absence of social information. Jones and DuVal (2019) found that copying effects were stronger in free-living populations, among sexually inexperienced females, and when social information countered an observer's innate preference for an attractive mate. Building on this, Davies et al. (2020) explicitly included tests of male mate choice copying and found little evidence of difference in the strength of this behaviour between the sexes. However, they did detect variations based on the focal taxonomic group and the type of experimental design employed.

Updating the evidence base through replication and quantitative research synthesis is fundamental to cumulative science (Nakagawa and Parker, 2015). In ecology and evolution, syntheses and reanalyses can alter conclusions about established effects and reduce their estimated magnitude (Nakagawa and Parker, 2015; Sánchez-Tójar et al., 2018; Yang et al., 2024). An updated meta-analysis challenged a canonical example of status signalling (Sánchez-Tójar et al., 2018), while corrections for selective reporting, including publication bias, substantially reduced meta-analytic effect-size estimates (Yang et al., 2024). We therefore expected an expanded and bias-corrected synthesis to recover a positive but smaller overall effect of mate choice copying than that reported in the original meta-analyses (Davies et al., 2020; Jones and DuVal, 2019).

In this meta-analysis, we used the datasets from Jones and DuVal (2019) and Davies et al. (2020) as our starting point. We updated the literature search, added newly identified studies, harmonised the effect-size data, and reanalysed the combined datasets using phylogenetically informed multilevel models. We also tested for small-study effects and time-lag patterns, applied corrections for publication bias, and conducted sensitivity analyses. Together, these steps allowed us to assess the evidence more rigorously while accounting for potential biases. Our specific research questions were as follows: (1) Does the overall average effect reported by the two previous meta-analyses remain supported? (2) How much do the magnitudes of the estimated effect sizes change when we add new evidence and update analytical methods? It should be noted that question 1 concerns the pooled average effect, rather than each individual moderator result reported by the original syntheses; we did not set out to retest those moderator findings individually.

## RESULTS

### Screening outcomes and dataset characteristics

We obtained a total of 69 new effect sizes from 29 studies for our meta-analysis update (Santiago et al., 2024; Belkina et al., 2021; Bierbach et al., 2011; Cirino et al., 2023; Clemente, 2012; Duffy et al., 2025; Harrison-Weiss, 2024; Kavaliers et al., 2019; 2022; Kniel et al., 2015; 2017; Kobayashi and Karino, 2026; MacLaren, 2024, 2019; Marcos, 2019; Miller, 2013; Mitchell et al., 2025; Moran et al., 2013; Nöbel et al., 2022a,b, 2023a,b, 2025; Nöbel and Kaufmann, 2025; Ophir, 2009; Pusiak et al., 2020; Silva, 2014; Witte et al., 2018; Ziege et al., 2012). Thirteen of these 29 studies (45%) were published in or before 2019 and therefore fell within the search window of the original meta-analyses; two of these 13 studies were included the dataset of Davies et al. (2020), leaving 11 studies (38% of our new studies) that fell in the search window but were not included in either original synthesis. The fact that our multilingual, grey-literature-inclusive strategy recovered this much pre-2019 evidence – including six theses (20.7% of the total) – suggests that the original searches were substantially less sensitive than their date ranges imply. The screening process and reasons for exclusion at the full-text screening stage are summarised in the PRISMA (Preferred Reporting Items for Systematic reviews and Meta-Analyses) flowchart (Fig. 1), with additional details provided in the lists of included and excluded studies (available in Tables S3 and S4). These studies reported mate choice copying trials for 15 species (Fig. 2; see Fig. S1 for the phylogeny combining the new and original meta-analysis data). *Drosophila* and *Poecilia* were the most studied genera in our sample, appearing in 11 and eight studies, respectively. These two genera accounted for 46 effect sizes (67% of the total).

**Fig. 1.**
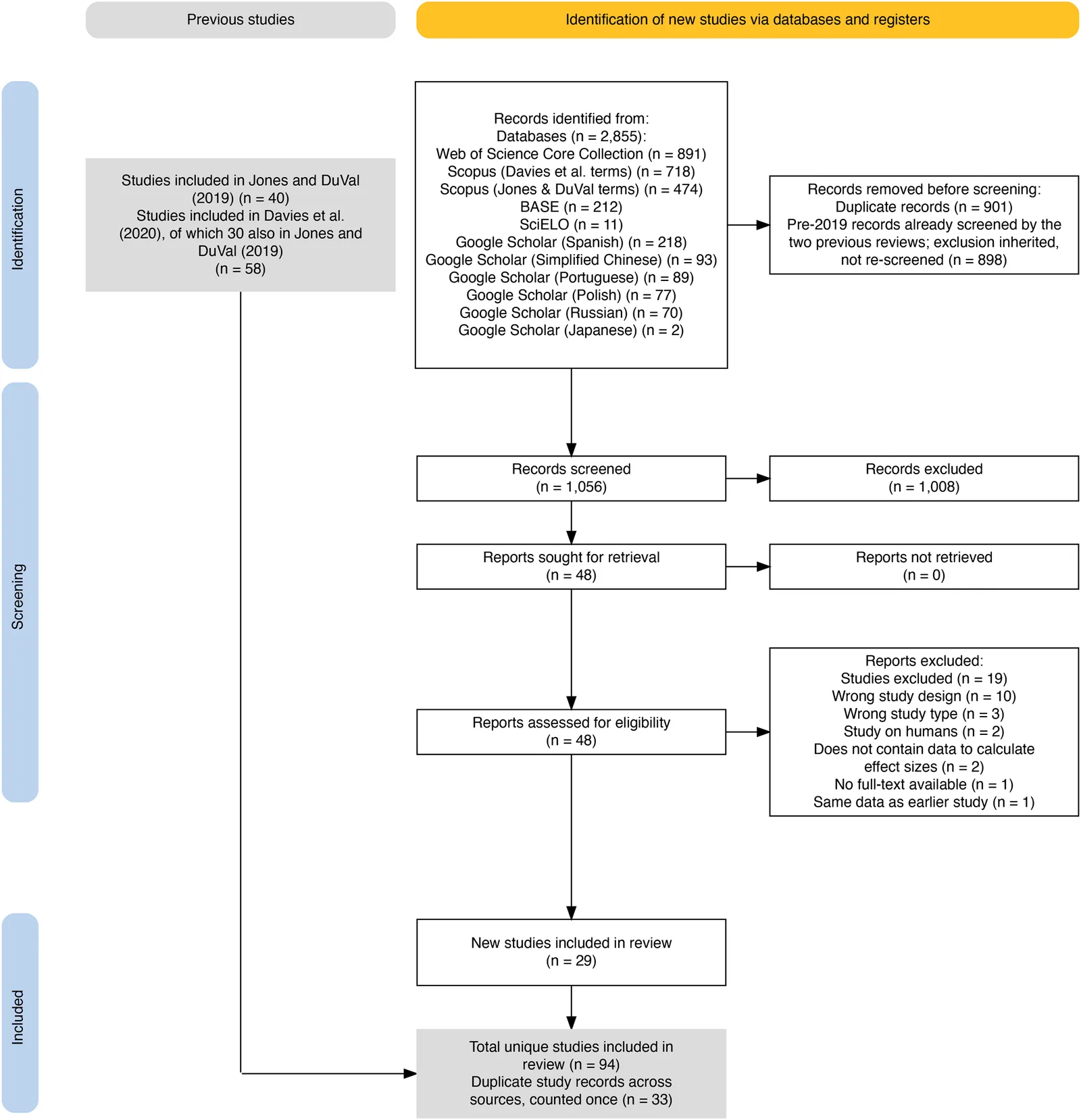
**PRISMA flow chart.** The diagram shows identification, screening, eligibility, and inclusion stages for the update of systematic reviews of the mate choice copying meta-analysis. Records retrieved are broken down by database and search platform; the number of studies shared between the datasets of Jones and DuVal (2019) and Davies et al. (2020) is stated in the ‘previous studies’ box, the pre-2019 records inherited from those two reviews are labelled with how they were handled, and the final box gives the number of unique primary studies together with the count of duplicate study records removed, so that the corpus can be reconciled directly from the diagram.

**Fig. 2.**
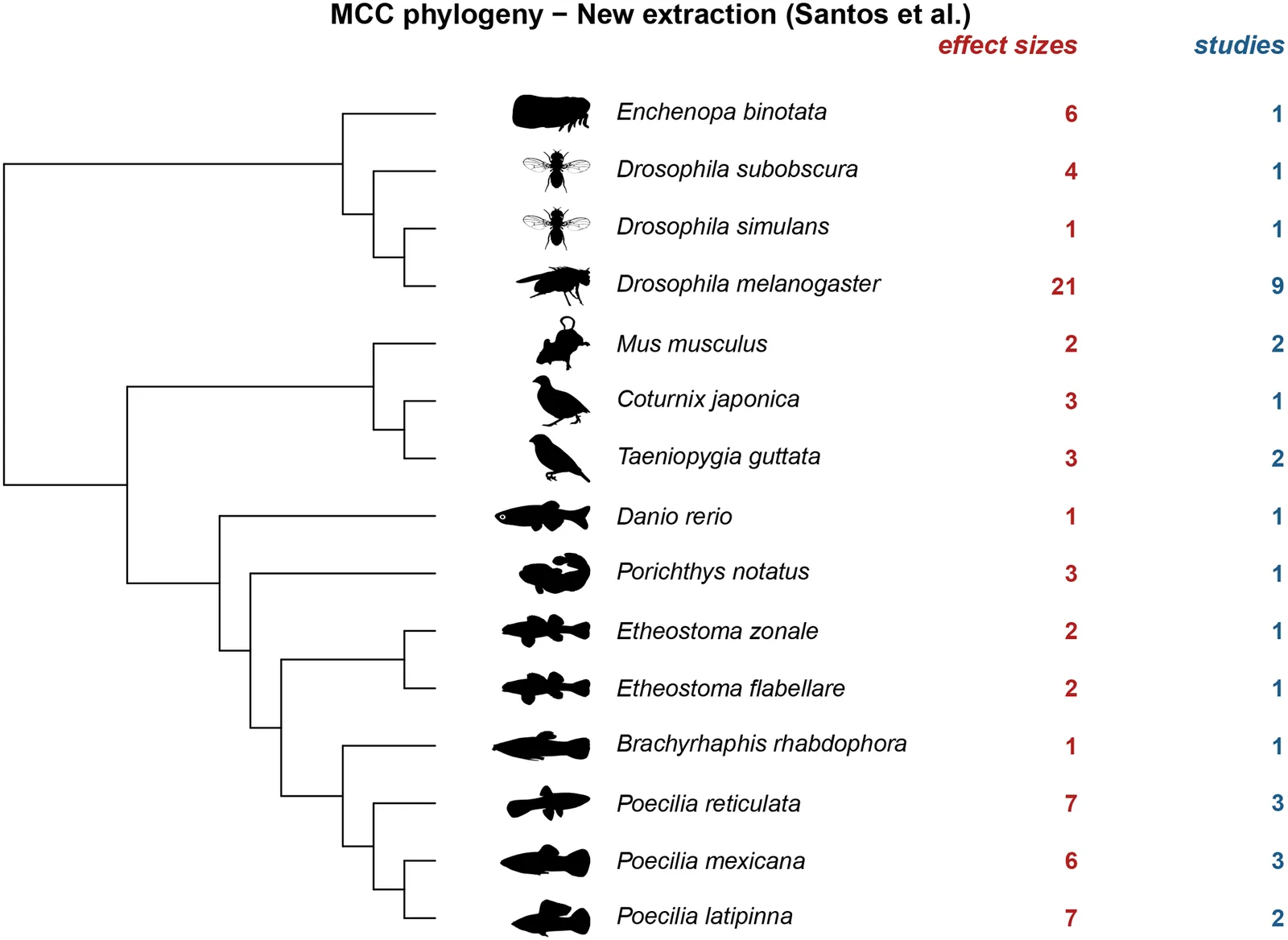
**Phylogenetic tree of the new extraction.** The phylogenetic tree of the 15 species added by this update, along with the number of effect sizes and the number of studies in which they are reported. Moran et al. (2013) used two *Etheostoma* species; thus, the ‘number of studies’ column total adds to 30, when in fact the actual number of studies included is 29. The tree for the full combined dataset of 33 species is given as Fig. S1.

Combining the new records with the two source datasets yielded a working corpus comprising 227 Hedges' *g* effect sizes from 87 studies and 172 log odds ratios (ORs) from 69 studies, spanning 33 species. Because the datasets of Jones and DuVal (2019) and Davies et al. (2020) overlap in their primary studies, the 40 studies carried over from the former and the 58 from the latter are not disjoint, and the 29 studies we added therefore do not sum with them to a count of unique primary studies. Resolving all three sources to distinct publications yields 94 unique primary studies. Thirty studies are common to the two source datasets; two of our 29 new studies were already in the dataset of Davies et al. (2020), having been recovered independently by our multilingual search and re-extracted from the primary reports; and one of the 40 study codes in Jones and DuVal (2019) duplicates another code within the same dataset. There is no overlap between our extraction and that of Jones and DuVal (2019), and no study appears in all three sources. These overlaps are shown explicitly in the PRISMA flow chart (Fig. 1), and the full study-level crosswalk, with the reason recorded for every accepted and rejected match, is provided in Tables S9 and S10.

### What is the overall evidence for mate choice copying?

Jones and DuVal (2019) reported a mean posterior OR for mate choice copying of 2.71 (95% credible interval: 1.60 to 4.80) in their original study. Using the new data, our mean overall OR is 1.46 [95% confidence interval (CI): 0.97 to 2.20]. Combining both datasets yielded an overall mean OR of 1.81 (95% CI: 1.34 to 2.45; Fig. 3B). However, this combined estimate is not directly comparable with the value of 2.71 reported by Jones and DuVal (2019) because their synthesis covered female observers only whereas ours includes both sexes. Restricting the combined OR dataset to female observers gave a mean OR of 1.81 (95% CI: 1.32 to 2.49; *k*=164 effect sizes from 66 studies), which is the estimate that can be set against theirs on like-for-like terms. The two estimates are effectively identical, so the difference from Jones and DuVal’s (2019) estimate of 2.71 is not an artefact of our having included male observers. All ORs are back-transformed from the log scale on which the models were fitted. With a 50:50 chance expectation, a mean OR of 1.81 corresponds to an observer choosing the socially favoured male on 64% of occasions; the original estimate of 2.71 corresponds to 73%, while our new data alone (OR=1.46) corresponds to 59%.

**Fig. 3.**
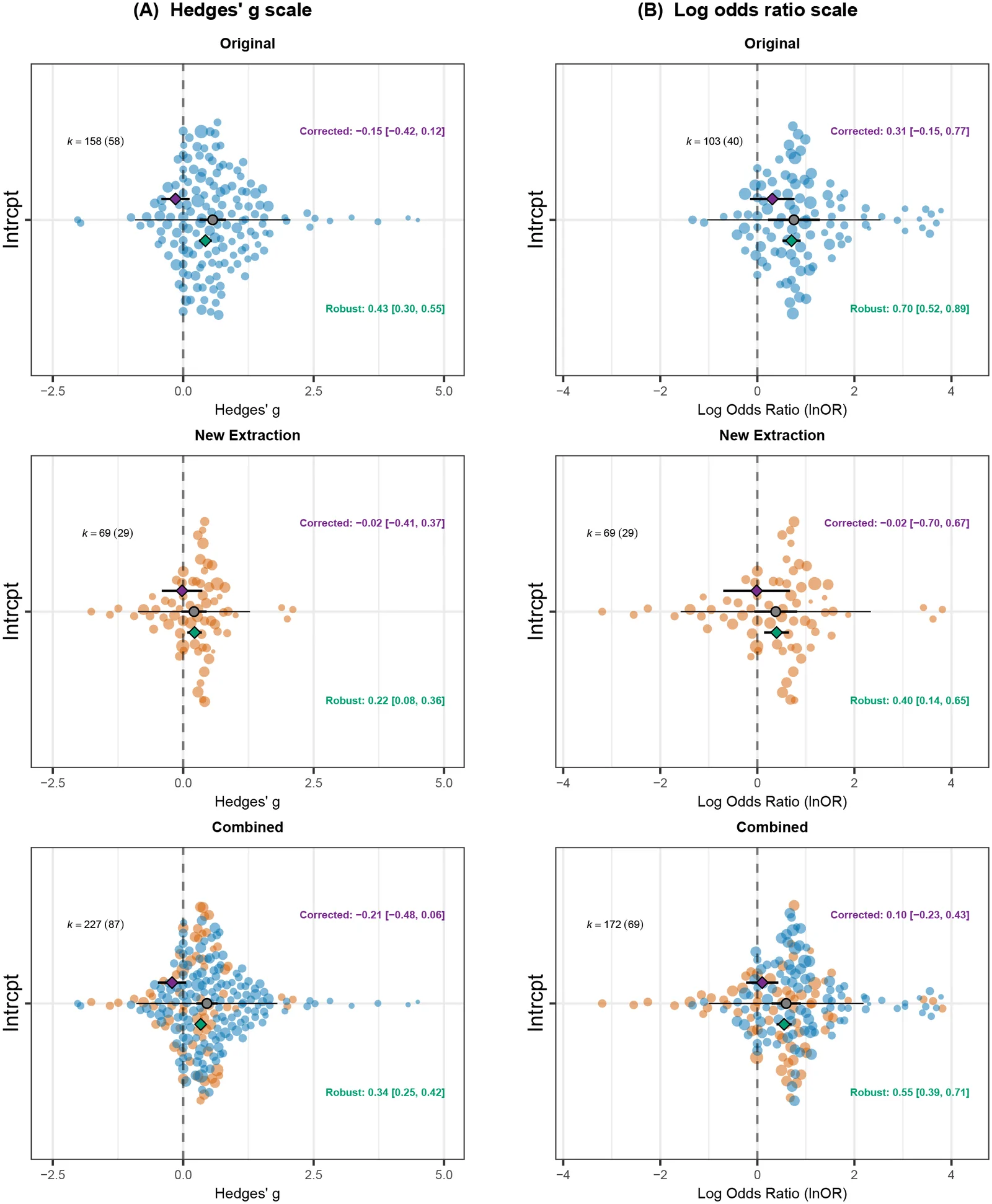
**Mean overall effect sizes on both scales.** (A) the Hedges' *g* scale and (B) the log OR scale. Within each column, the three rows are the original, new (this study) and combined (original+new) datasets, and each row presents three meta-analytic estimates: bias corrected (annotated ‘Corrected’; Nakagawa; purple diamond), uncorrected (grey circle), and bias robust (annotated ‘Robust’; Yang; green diamond). Thick horizontal lines represent 95% CIs, and thin horizontal lines represent 95% prediction intervals. The points in the centre of each thick line indicate the average meta-analytic effect size. *k* is the number of effect sizes used to estimate the statistics, followed by the number of studies in the brackets. Each bubble is an individual effect size; the bubble area is proportional to its precision (1/s.e.).

Davies et al. (2020) reported a mean overall Hedges' *d* of 0.58 (95% CI: 0.34 to 0.83), conventionally described as a ‘moderate’ effect (*sensu* Cohen), although such benchmarks are arbitrary and poorly calibrated to behavioural and evolutionary data (Nakagawa and Cuthill, 2007). Our new data had a mean overall Hedges' *g* of 0.21 (95% CI: −0.02 to 0.43), a ‘small’ effect on the same scale. The combined mean overall Hedges' *g* is 0.45 (95% CI: 0.25 to 0.66; Fig. 3A).

Heterogeneity was substantial in all models. *I*^2^ – the proportion of observed variance not attributable to sampling error – exceeded 74%, and the total among-effect-size variance (*τ*^2^) was 0.43 for Hedges' *g* and 0.58 for log OR, giving 95% prediction intervals of −0.90 to 1.81 and −1.00 to 2.19, respectively, for a new study (for details, see Table 1). We report both values because *I*^2^ is a proportion and therefore depends on the precision of the contributing studies, whereas *τ*^2^ and the prediction interval express heterogeneity on the scale of the effect size itself. More specifically, study ID (representing the between-study effect) accounted for up to 10.5% across models, whereas observation ID (representing the within-study effect) accounted for most of the heterogeneity on both scales (69.8% for Hedges' *g*, 45.4% for log OR). Species identity (the non-phylogenetic species effect) contributed substantially to the log OR scale (23.9%), but only minimally to the Hedges' *g* scale (1.9%). Phylogenetic relatedness explained little heterogeneity across models (*I*^2^≤7.1%).

**Table 1.** Partial *I*^2^ (%) for each random effect across the uncorrected multilevel models

Analysis	Total	Study	Effect size (residual)	Species (non-phylogenetic)	Phylogeny
Hedges' *g* – new only	80.8	0.0	51.3	29.4	0.0
Hedges' *g* – original only	85.8	4.1	77.1	0.0	4.6
Hedges' *g* – combined	85.5	10.5	69.8	1.9	3.3
Log OR – new only	80.8	0.0	51.3	29.5	0.0
Log OR – original only	75.9	0.0	23.2	45.6	7.1
Log OR – combined	74.7	4.3	45.4	23.9	1.1

Total is the overall proportion of variance due to heterogeneity (versus sampling error); the remaining columns partition that heterogeneity among the study, residual effect-size, non-phylogenetic species, and phylogenetic components.

### Uni-moderator analyses of the taxonomic group

We conducted uni-moderator analyses of Hedges' *g* and log-ORs to examine how mate choice copying varied among taxonomic groups (arthropods, fish, and other vertebrates). All estimated means were positive across the three groups in both models using the combined datasets (Fig. 4). On the Hedges' *g* scale, the point estimates increased from arthropods (*g*=0.25, 95% CI: 0.03 to 0.47) to fish (*g*=0.50, 95% CI: 0.30 to 0.70) and to the other vertebrates, i.e. birds and mammals (*g*=0.70, 95% CI: 0.35 to 1.05). Total heterogeneity was high (94.3%) and the among-group test approached statistical significance (*F*_2,27_=3.00, *P*=0.066). On the log OR scale the ranking differed, with fish being the highest (log OR=0.82, 95% CI: 0.37 to 1.26), followed by the other vertebrates (log OR=0.61, 95% CI: −0.04 to 1.25) and then arthropods (log OR=0.33, 95% CI: −0.13 to 0.80). Total heterogeneity was again high (89.5%), and differences among groups were not significant (*F*_2,25_=1.17, *P*=0.327). Residual variance was itself group specific and roughly four times larger in fish (*τ*^2^=0.53 for Hedges' *g*, 1.04 for log ORs) than in arthropods (0.12 and 0.15). Because the two scales give different rankings and neither omnibus test is significant, we do not interpret the apparent gradient. The taxonomic group is not a new moderator. Davies et al. (2020) also included this moderator , and it was one of only two of their ten moderators to reach significance (*Q_M_*=8.57, *P*=0.04), with copying being strongest in mammals (*k*=6 effect sizes), followed by birds (*k*=16) and fish (*k*=103), and weakest in arthropods (*k*=33). On the Hedges' *g* scale, our combined analysis – which pools birds and mammals with other vertebrates – recovers the same ordering, but the contrast is no longer statistically supported once the new evidence is added (*F*_2,27_=3.00, *P*=0.066), and on the log OR scale, the ordering does not hold. Our contribution here is therefore to revisit an existing result on an expanded dataset rather than to test a new question, and the substantive finding is that the taxonomic contrast weakens and becomes scale dependent.

**Fig. 4.**
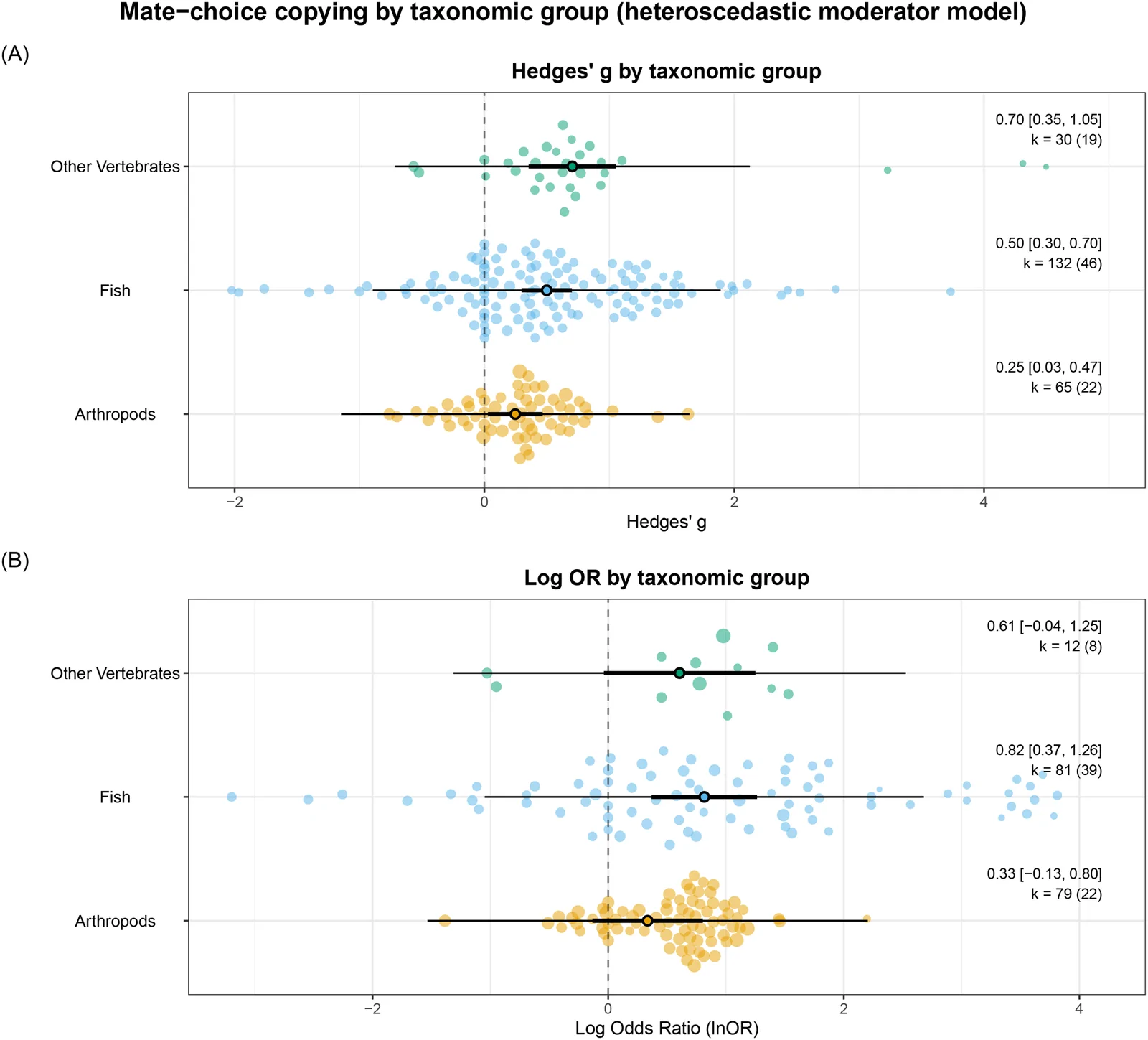
**Taxon moderator effect sizes.** (A) the Hedges' *g* scale and (B) the log OR scale. All panels present three meta-analytic estimates: other vertebrates (birds and mammals; green), fish (blue), and arthropods (yellow). Thick horizontal lines represent 95% CIs, and thin horizontal lines represent 95% prediction intervals. The points in the centre of each thick line indicate the average meta-analytic effect size. *k* is the number of effect sizes used to estimate the statistics, followed by the number of studies in brackets.

### Exploratory uni-moderator analyses of biological moderators

No moderator explained a detectable share of the heterogeneity on either scale (all *F*-tests, *P*≥0.17; total *I*^2^ remained above 80%). Three contrasts are nonetheless worth noting. First, the strength of copying did not significantly differ between the two copying mechanisms: generalised and individual copying were essentially identical in the combined Hedges' *g* data (0.43 versus 0.46; *F*_1,225_=0.05, *P*=0.82), and in the new extraction, the generalised estimate was, if anything, marginally larger (Hedges' *g*=0.26 versus 0.16; log OR=0.48 versus 0.30; both *P*>0.5). Second, whereas Jones and DuVal (2019) reported stronger copying among virgin observers, our new extraction showed the opposite ordering (Hedges' *g*=0.09 for virgin versus 0.36 for non-virgin observers; *F*_1,27_=1.61, *P*=0.22), and the significant difference disappeared when the new data were pooled with the original binary data (OR=1.75 versus 1.91; *F*_1,170_=0.13, *P*=0.72). Third, male observers copied at least as strongly as females, both in the new extraction (Hedges' *g*=0.51 for males versus 0.16 for females; log OR=0.94 versus 0.30; *F*_1,67_=1.95 and 1.94, respectively, *P*=0.17 on both scales) and in the combined Hedges' *g* data (0.51 versus 0.45; *F*_1,225_=0.17, *P*=0.68). The male level is based on only eight effect sizes from five studies in the new extraction, however, so this is a weak test: the sensible reading is that there is no evidence of a sex difference, not that the sexes are equivalent. No contrast was statistically significant on either scale. Estimates for every biological moderator level, on both scales, are shown in Fig. 5; full diagnostics, model summaries and between-level contrasts are provided in Tables S5–S8.

**Fig. 5.**
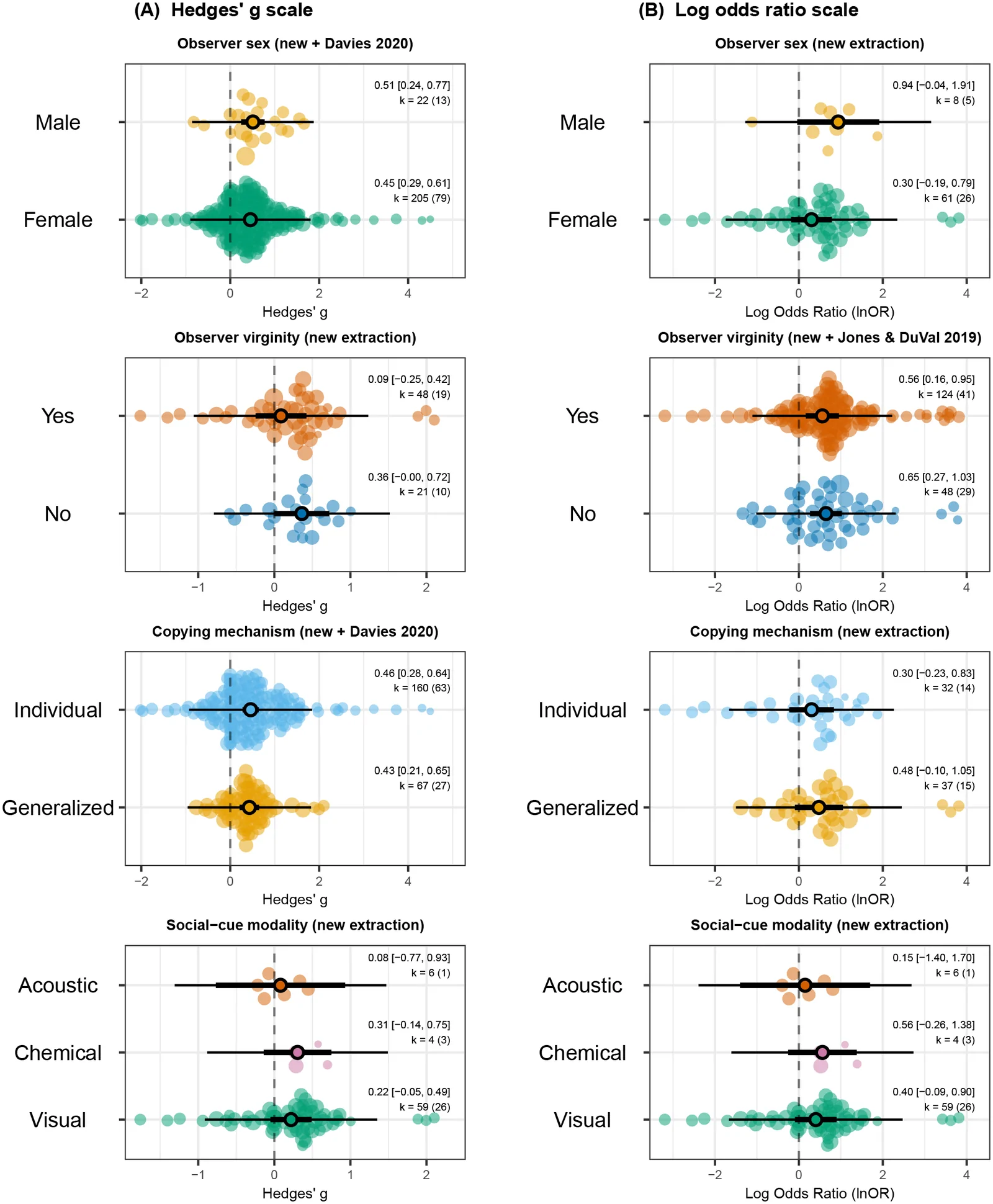
**Exploratory biological moderators.** Orchard plots of the estimated mean effect for each level of the biological moderators extracted during data collection – observer sex, observer mating status (virgin versus non-virgin), copying mechanism (generalised versus individual), and social cue modality – on (A) the Hedges' *g* scale and (B) the log OR scale. Each panel uses the widest dataset available for that moderator and scale, stated in the sub-panel heading: observer sex and copying mechanism harmonise with the data of Davies et al. (2020) on the Hedges' *g* scale, observer mating status harmonise with the data of Jones and DuVal (2019) on the log OR scale, and the remaining models are fitted to the new extraction alone. Thick horizontal lines represent 95% CIs, and thin horizontal lines 95% prediction intervals; the point in the centre of each thick line is the marginalised mean for that level. *k* is the number of effect sizes, followed by the number of studies in brackets. The bubble area is proportional to precision (1/s.e.).

### Small-study effects, time-lag patterns, and sensitivity analyses

We found strong evidence of small-study effects, and mixed evidence of time-lag bias, using the combined dataset. Funnel plots based on model residuals showed clear asymmetry for both types of effect size (Figs S2 and S3). Egger's-type multilevel regression slopes on the square root of the sampling variance were positive and statistically significant for both datasets (log OR: 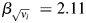, 95% CI: 1.23 to 2.99; Hedges' *g*: 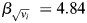, 95% CI: 3.59 to 6.09; Fig. 6). For the OR dataset, publication year was negatively associated with effect-size magnitude (*β*_year_=−0.028, 95% CI: −0.048 to −0.007; Fig. 6B), whereas for the Hedges' *g* dataset, there was little evidence of such a decline (*β*_year_=−0.005, 95% CI: −0.018 to 0.008; Fig. 6A).

**Fig. 6.**
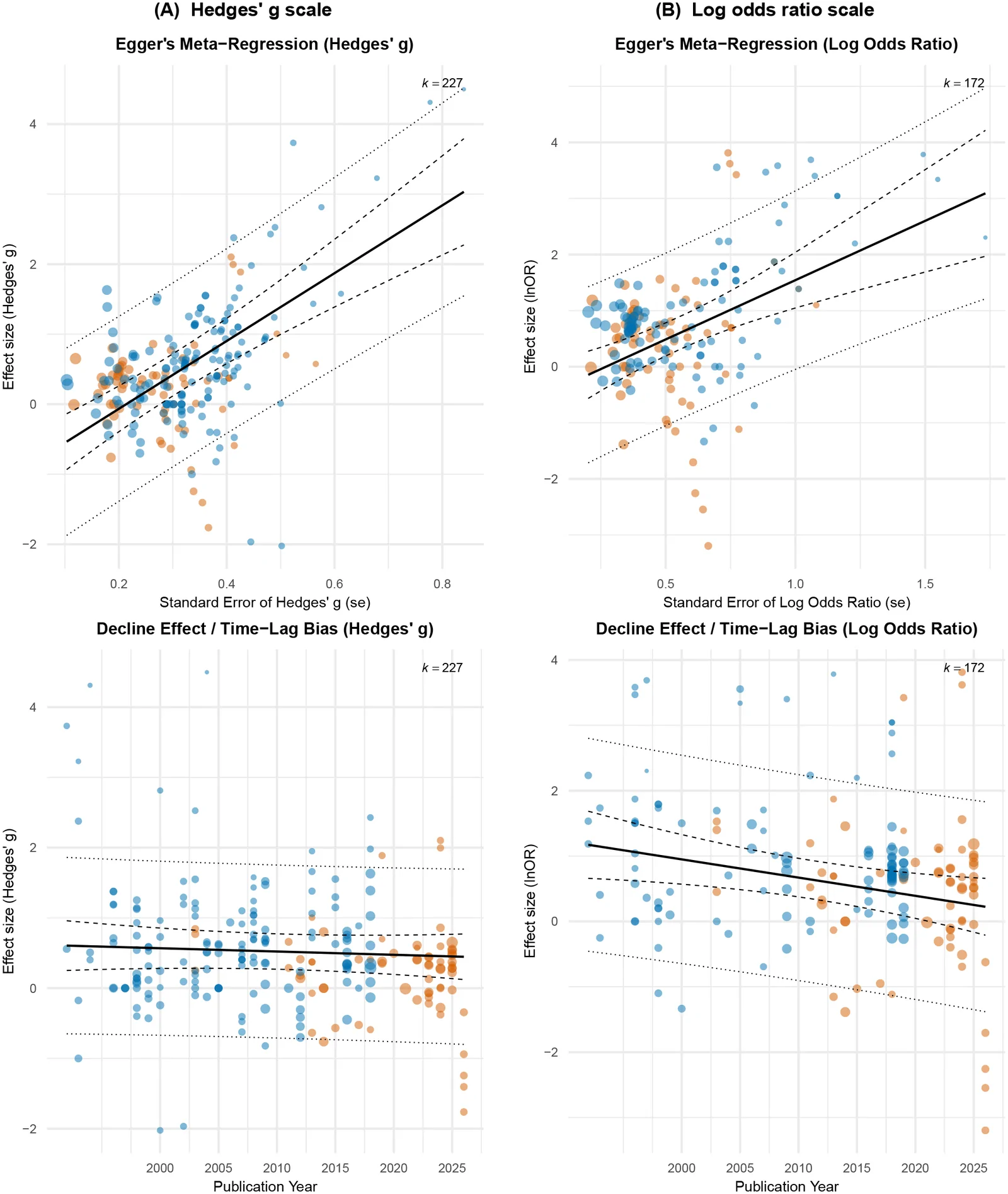
**Publication-bias diagnostics.** (A) The Hedges' *g* scale and (B) the log OR scale. Within each column, the upper panel shows the association between effect sizes and the square root of the sampling variance (Egger's meta-regression) and the lower panel the association with publication year (time-lag bias). Point size reflects precision; original data (blue) and new data (orange); solid lines show fitted meta-regressions, and dashed and dotted lines indicate 95% confidence and prediction intervals, respectively.

The two correction frameworks gave divergent adjusted means, both of which were smaller than the uncorrected estimates. On the Hedges' *g* scale, the bias-corrected intercept (Nakagawa et al., 2022) decreased to −0.21 (95% CI: −0.48 to 0.06), a mean indistinguishable from zero, whereas the bias-robust estimator (Yang et al., 2024) retained a positive but reduced mean of 0.34 (95% CI: 0.25 to 0.42). The OR scale showed the same pattern: the bias-corrected mean approached the null at a log OR of 0.10 (95% CI: −0.23 to 0.43, i.e. OR=1.11), while the bias-robust mean remained clearly positive at a log OR of 0.55 (95% CI: 0.39 to 0.71; OR=1.73). Both corrected means are shown alongside the uncorrected estimate in Fig. 3.

Leave-one-out analyses confirmed the robustness of both pooled estimates. Sequential exclusion of individual studies yielded consistent means, with every CI overlapping the full-sample estimate and no single study exerting disproportionate influence; the largest shift was 0.02 Hedges' *g* units and 0.04 log-odds units (Figs S4 and S5). The results were also robust to leave-one-species-out analyses, which remove entire species despite the uneven phylogenetic sampling, with all overall effect sizes remaining significantly positive (Figs S6 and S7). Here, the largest shift was 0.04 Hedges' *g* units (omitting *Poecilia latipinna*) and 0.07 log-odds units. Removing *Poecilia reticulata* – the most-studied and strongest-copying species, which alone contributes nearly a quarter of the continuous effect sizes – nudged the Hedges' *g* mean towards zero, as expected, but did not change the sign or significance of either synthesis.

The combined estimates also showed little sensitivity to the contentious effect sizes on either scale. The pooled Hedges' *g* value of 0.45 (95% CI: 0.25 to 0.66, *k*=227) decreased to 0.41 (95% CI: 0.22 to 0.60, *k*=213) when the flagged studies were removed, and to 0.42 (95% CI: 0.23 to 0.60, *k*=225) when they were corrected, remaining significant throughout (Fig. S8). Similarly, the OR synthesis remained stable, with the pooled OR decreasing from 1.81 (95% CI: 1.34 to 2.45, *k*=172) to 1.77 (95% CI: 1.32 to 2.38, *k*=156) under leave-one-out analyses and then increasing again to 1.79 (95% CI: 1.36 to 2.34, *k*=169) under correction (Fig. S9).

## DISCUSSION

By updating the datasets from the two original meta-analyses and reanalysing them within a unified, phylogenetically informed multilevel framework – 227 Hedges' *g* effect sizes from 87 studies and 172 log ORs from 69 studies, spanning 33 species – we can now answer the two questions we set out with. First, the qualitative conclusion of both foundational syntheses persists: across an expanded, multilingual, and grey-literature-inclusive search, observers experimentally exposed to social information were, on average, more likely to favour the associated mate, and the pooled effect remained positive and statistically significant on both the OR and standardised mean difference scales. Second, the effect is smaller than previously implied – roughly half the original magnitude when the new evidence is considered on its own, with CIs that span zero, and, under the more conservative bias correction, an adjusted mean close to zero. Mate choice copying therefore occurs across a broad range of taxa but is more modest and more context dependent than Jones and DuVal (2019) and Davies et al. (2020) implied – a pattern that is itself informative and that we explore below.

### Mate choice copying as social learning and its consequences for sexual selection

Theory frames mate choice copying as one expression of a general capacity to use social information, alongside socially guided foraging, habitat selection, and predator avoidance (Danchin et al., 2018; Galef and Laland, 2005; Valone and Templeton, 2002), with a central prediction that such information supplements, rather than overrides, an observer's own assessment of a prospective mate (Vakirtzis, 2011; Uehara et al., 2005). A reliably positive yet modest average effect is what that prediction implies, and it reframes the biological question from whether animals copy to when they do so. The theory does not predict indiscriminate copying: social learning strategies such as ‘copy when uncertain’ and ‘copy when young or inexperienced’ specify the narrow conditions under which relying on others outperforms personal information (Laland, 2004; Galef and Laland, 2005). Seen this way, the recent, well-powered studies that report weak, null, or context-restricted copying (Nöbel et al., 2023b; Belkina et al., 2021; Pusiak et al., 2020) are not evidence against the phenomenon but, possibly, evidence that, as theory predicts, it is expressed selectively – animals discount or reverse the behaviour when public information is unreliable, when demonstrators are sperm limited, or when individual differences in personality intervene (Loyau et al., 2012; Dugatkin et al., 2003; Nöbel et al., 2022c).

This conditional view also reframes the most prominent feature of our results – large and largely unexplained heterogeneity – as biological signal rather than statistical noise. Variation accumulated within studies and among species, rather than between laboratories or across the phylogeny, which is what we would expect if the expression of copying is tuned to the observer's experience, the reliability and consistency of the social information, and the local ecological context. Jones and DuVal (2019) found exactly such conditionality, with stronger copying among inexperienced (virgin) females; our updated data, however, did not reproduce this virginity effect and, in the new studies, tended to reverse it. The candidate moderators we fitted – observer sex, copying mechanism, cue modality and observer mating status (demonstrator age showed no variation and could not be analysed) – were explored only in *post hoc*, non-pre-registered analyses and explained no detectable share of the heterogeneity, consistent with the relevant variation residing at finer or more interactive scales than our coding captured. That copying occurs even in animals lacking complex sociality, most notably *Drosophila*, further suggests that it taps domain-general learning mechanisms shared widely across animals rather than lineage-specific cognition (Bridges and Chittka, 2019; Danchin et al., 2018). This interpretation is consistent with the negligible phylogenetic signal that we recovered.

The location of the heterogeneity differs between the two scales. Species identity absorbed 23.9% of it on the log OR scale but only 1.9% on the Hedges' *g* scale, while phylogeny absorbed almost none on either (≤3.5%; Table 1). This difference reflects the dataset composition rather than biological factors: species accounted for 29% of the heterogeneity in our new extraction and 45.6% in the binary data of Jones and DuVal (2019), but for none at all in the continuous data of Davies et al. (2020). Two points follow. Where species variance is detectable, it is not organised by relatedness, which points to mating system, ecology, and assay tradition rather than to lineage-specific cognition. However, since *Drosophila* and *Poecilia* account for two-thirds of all effect sizes, ‘among-species’ variance is partly attributable to variance among the few laboratories that study these species; with the taxonomic gradient reversing between scales and neither omnibus test being significant, we do not treat it as established.

Much of the remaining variance likely lies in design features. Two of these – experimental design (before and after versus no pretest) and choice outcome (mating versus association time) – were extracted during data collection, but their levels are confounded with species and assay tradition in the combined corpus, and neither was pre-registered as a moderator, so we did not fit them. Others were not coded at all: live versus video demonstrators and – for 22 of the 38 binary effect sizes – whether a control group existed or a chance expectation stood in for one. These axes plausibly generate more variance than the biological moderators we tested, which would explain why none of the latter absorbed a detectable share.

Variation also enters through the data extraction process itself. The two source syntheses report overlapping studies on different metrics, and for seven of them, the values disagreed by more than 0.5 Hedges' *g* units (when converted to the same scale). Re-extraction from the primary sources resolved every one towards the ORs of Jones and DuVal: the continuous extraction had variously captured a different construct (e.g. association time and body size), doubled paired-sample sizes, produced artefacts of degenerate 2×2 tables, and in one case reversed the sign, turning a reported absence of copying into apparent evidence for it. The binary extraction had its own issues, overcounting effect sizes in two studies. Our estimates are robust to how these values are handled (supplementary information), but this issue is important: much of what reads as biological disagreement between the two syntheses was extraction disagreement – an argument for cross-validating source data whenever meta-analyses are combined and/or updated.

Finally, one distinction among our exploratory moderators carries particular weight in terms of evolutionary consequences: whether observers copy in a generalised or an individual fashion. Only generalised copying – the acquisition of a preference for a male phenotype or trait rather than for one specific individual – can propagate through a population, bias the targets of sexual selection, and initiate the cultural transmission of mating preferences, although theory disagrees on how strongly copying ultimately accelerates or constrains sexual selection (Varela et al., 2018); a preference attached to one individual, by contrast, ends with the demonstrated male. It is worth separating this evolutionary distinction from the experimental one it is inferred from. Our moderator codes the design of a trial, not the decision process of the observer: generalised designs present observers with different target individuals sharing a trait, whereas individual designs re-present the same targets. The two are not equally informative about the evolutionary question – an individual design cannot distinguish a preference generalised to the trait from one attached to the specific individual or from other latent cues correlated with it but neither is the more rigorous test, and individual designs are often the only feasible option for a given system. Our data are therefore consistent with, rather than a direct test of, the generalised form being robustly expressed: copying strength was statistically indistinguishable between the two designs and, if anything, marginally stronger under generalised designs in the new extraction. A design that manipulates the target trait explicitly and pre-registers the generalisation test would generate valuable information, and we regard this as a high priority for future work.

### Limitations, knowledge gaps, and future opportunities

Several features of our approach constrain the strength of these inferences. Because the two source meta-analyses reported different metrics, placing all studies on a common scale required approximate conversions that assume an underlying logistic distribution: in our new extraction alone, 38 Hedges' *g* values were converted from ORs and 31 log ORs from Hedges' *g*. 22 of the 38 binary effect sizes had no separate control group and relied on an equal-allocation chance expectation, which likely understates their sampling variance. Much of our effect-size data were extracted from published figures; the agreement between our extraction routine and ‘metaDigitise’ on a validation subset was close (mean signed *z*-score=−0.10), but figure extraction remains a source of error. Finally, several moderator levels were sparsely sampled, and none of the biological moderator analyses were pre-registered, so those results are exploratory.

Three features of the evidence base temper these conclusions and set the agenda for future work. First, we found strong small-study effects on both scales; under the bias-corrected intercept (Nakagawa et al., 2022), the adjusted mean collapsed towards zero, whereas the bias-robust estimator (Yang et al., 2024) retained a positive but reduced signal. A cautious interpretation is that the true effect very likely lies below the naïve pooled estimate and that a much smaller effect cannot be excluded on present evidence. Second, for the binary outcomes, the effect-size magnitude declined with publication year, echoing the familiar pattern in which early, large effects are gradually tempered by more rigorous and better-powered designs (Jennions and Møller, 2002; Koricheva and Kulinskaya, 2019); several of the most recent studies in our sample report weak or null copying (Belkina et al., 2021; Cirino et al., 2023; Pusiak et al., 2020; Nöbel et al., 2023b). Third, primary studies quantify copying in incompatible ways, forcing approximate conversions and leaving a handful of studies whose values disagreed materially between datasets. Although our conclusions proved robust to how these contentious values were handled, the broader lesson is methodological: the field would benefit from registered reports, the routine publication of null and small-sample results, and minimum reporting standards – group means, s.d.s, sample sizes, and raw choice counts – so that future updates are based on less-censored, directly comparable data (Davies et al., 2020).

A further gap concerns taxonomic breadth. Although our combined sample spanned 33 species across arthropods, fish, and other vertebrates, the evidence remains concentrated in two model genera: *Drosophila* and *Poecilia* together contributed roughly two-thirds of all effect sizes. This is precisely the situation in which a sample concentrated in two genera is least informative and in which broader taxonomic coverage would be most valuable. Reassuringly, the uni-moderator analyses returned positive means in all three taxonomic groups on both scales, with only weak support for among-group differences, so we treat the apparent gradient cautiously. The taxonomic contrast that detected by Davies et al. (2020) (*Q_M_*=8.57, *P*=0.04) was largely driven by mammals, for which they had six effect sizes from two species. On the expanded corpus, the same ordering survives on the Hedges' *g* scale, but the contrast is no longer significant, and it does not hold on the log OR scale. We read this as evidence that the earlier taxonomic result was not robust to the addition of new evidence, rather than as a new taxonomic result of our own. Recalculating the pooled mean from those group means with the three groups weighted equally – an approximation to balanced taxonomic coverage – shifts it by less than 0.04 Hedges' *g* units and by less than 0.01 log OR units, so the estimates are not an artefact of the sample's concentration in arthropods. Invertebrates, amphibians, reptiles, non-poeciliid fishes, and a broader range of birds and mammals remain effectively absent. Expanding the phylogenetic spread of the evidence would both test the generality of mate choice copying and improve phylogenetic comparative tests that, which currently have little signal to work with.

### Conclusions

Mate choice copying is a quantifiable and taxonomically widespread behaviour: after incorporating seven additional years of evidence and analysing it with contemporary phylogenetic multilevel methods, we still recover a positive and significant overall effect on both the OR and standardised-mean-difference scales. At the same time, the updated picture is more tempered than the original meta-analyses suggested. The pooled effect is moderate at best; is surrounded by substantial heterogeneity that our models could not attribute to study, taxon, or phylogeny; and bears clear fingerprints of small-study and selective-reporting bias that, under the more conservative correction, draw the estimate close to zero. The qualitative claims of Jones and DuVal (2019) and Davies et al. (2020) hold, but their estimated magnitudes were probably optimistic. Realising the promise of mate choice copying as a driver of sexual selection and cultural evolution will require broader taxonomic sampling, mechanism-focused and pre-registered tests of the moderators that potentially influence its considerable heterogeneity, and a publication culture that faithfully reports null findings. Finally, we present this update as a model for how meta-analyses in ecology and evolution can be revisited – combining new evidence with the original data, harmonising incompatible effect-size metrics, and stress-testing conclusions against publication bias and contentious extractions.

## MATERIALS AND METHODS

We pre-registered our methods and planned analyses before data extraction and analysis on the Open Science Framework (https://osf.io/vfahk/overview; Santos et al., 2026). Throughout our review and analysis process to guide the reporting, we followed the PRISMA-EcoEvo (PRISMA in Ecology and Evolutionary biology) guidelines (O'Dea et al., 2021; Table S2). We report author contributions to each methodological step using the Method Reporting with Initials for Transparency (Nakagawa et al., 2023), providing author initials in-line throughout the Materials and Methods section. Where initials might be ambiguous, we disambiguate by surname: A. Mizuno (Ayumi Mizuno) and A. Milenović (Aleksandra Milenović); C. Schneider (Cassidy Schneider) and C. Sosiak (Christine Sosiak).

### Additions and deviations

We made a few changes to elements of our pre-registered protocol. We implemented only the search strategy described under question 2 in the pre-registered protocol because the more comprehensive, multilingual, grey-literature-inclusive strategy fully encompassed the narrower search, making the additional strategy redundant. We included the same studies as in the earlier meta-analyses (these were automatically retained in our dataset), as well as new studies from all periods that were retrieved in our searches, as retention of the original studies is a key element of an update rather than an entirely new synthesis and allowed for direct reconciliation of old and new estimates on a common scale. We did not extract *de novo* effect sizes from original meta-analyses but instead cross-checked effect sizes present in both original meta-analyses; cross-checking (rather than re-extracting) enables us to flag discrepancies between the two source datasets while avoiding the risk of introducing new extraction error.

### Eligibility criteria and search protocols

We used the PECOS (Population, Exposure, Comparator, Outcomes, and Study design) framework (Richardson et al., 1995; Foo et al., 2021) to define a single set of eligibility criteria, applied to both the literature search and the two screening stages, following our pre-registered protocol (Santos et al., 2026) and the criteria outlined by Jones and DuVal (2019) and Davies et al. (2020).

‘Population’ includes non-human, sexually mature female or male individuals from wild or captive populations of multicellular animals. Studies that experimentally manipulated sexual maturity or prior mating experience were eligible provided the focal individuals were mature at the time of testing.

‘Exposure’ refers to tests of the audience effect during mate choice trials; that is, the observation of a social cue (e.g. a female near or copulating with a male). The social cue could be the presence of a model or demonstrator individual of the same sex as the focal individual; a chemical, visual, or auditory cue; or an observed copulation.

A ‘comparator’ is a group (sample) of females or males making a choice of mate without social cues or with neutral/conflicting cues. For instance, a no-information control comprises individuals tested under identical conditions but with no model present or with the model hidden behind an opaque partition – so any preference reflects chance/baseline rather than social information. Pre-demonstration preference comparators and comparisons with an explicit chance expectation were also considered.

The ‘outcome’ refers to the mating preference of the focal/observer individual. Studies had to report, for each focal individual, either a discrete mate choice or a continuous measure of preference. Examples include preference-zone time, defined as the number of seconds that the observer spent within a specific distance of a target individual after the demonstration; a social learning index, calculated to quantify the bias towards the phenotype chosen by the observer; and copulation choice, defined as the phenotype the focal/observer individual chose to mate with when physical access was allowed.

The ‘study design’ comprised empirical experimental studies that expose focal/observer individuals to opposite-sex mates (with and without an audience effect), in which one focal or observer individual chooses between two individuals of the opposite sex, one of which – the target – was associated with stimuli from a model or demonstrator.

#### Additional restrictions

Beyond the PECOS criteria, studies were eligible only if (i) they were reported in English, Japanese, Polish, Portuguese, Russian, Simplified or Traditional Chinese, or Spanish and (ii) they reported sufficient statistical information to calculate an effect size – group means with s.d.s. or standard errors (s.e.) and sample sizes, raw choice counts, or a convertible test statistic. Effects reported only as *F*-statistics, *χ*^2^ values, or generalised linear mixed-model outputs were not convertible and were excluded (Table S4).

We conducted a literature search across five databases and platforms, and no others: Web of Science Core Collection, Scopus, Google Scholar (for non-English studies), the Scientific Electronic Library Online (SciELO), and the Bielefeld Academic Search Engine (for theses and dissertations, i.e. grey literature). Search strings, access dates and the number of records retrieved from each source are given in Table S1. We designed the search strings based on a pilot search-string strategy to assess sensitivity, feasibility, and transparency using the benchmarking/relative recall approach (Lagisz et al., 2025). We did not restrict the database searches by publication year, language, subject area, or document type. The strings were translated for searches in non-English languages, and search results were assessed by reviewers with expertise in the respective languages: A. Mizuno for Japanese, M.L. for Polish and Russian, E.S.A.S. for Portuguese, H.Q. for Simplified/Traditional Chinese, and S.O. for Spanish. We did not limit the number of records returned by the Google Scholar searches in each language; the records were sorted by relevance. We conducted Google Scholar searches using the software ‘Publish or Perish, v8.19’ (Harzing, 2007). Because many retrieved records, especially non-English ones, carried truncated abstracts, we recovered abstracts with ‘ris-enrich’ (Santos, 2026 preprint) before conducting the title-and-abstract screening.

### Study selection and screening

After the literature search, we deduplicated records and proceeded to our two-stage screening (with participation by all authors), following the procedures described in our pre-registered protocol. In cases of disagreement between the reviewers, discrepancies were discussed and resolved to reach a consensus. The screening process and results are shown in the PRISMA flow chart (Fig. 1; built from a single table of counts with the ‘PRISMA2020’ R package; Haddaway et al., 2022). Of the 2855 records retrieved, 901 were duplicates, and a further 898 were pre-2019 records that had already been screened by Jones and DuVal (2019) and Davies et al. (2020) within their own search windows. For those 898, we inherited the original reviews' exclusion decisions rather than readjudicating them, and the flow chart labels them accordingly; 1056 records therefore entered our own two-stage screening. This is a deliberate limitation of the record: to the extent that the earlier searches were less sensitive than their date ranges imply – as our results suggest, given that 11 of our 29 new studies are pre-2019 papers not included in either original synthesis – inheriting their exclusions carries that limitation forward for the records they had already seen.

### Data collection

We extracted three types of information from each study. First, we collected citation information, such as title, author name, and publication year. Second, we collected the details of each experiment within studies: the species studied, type of experimental design (before and after or no pretest), type of copying mechanism (generalised: across trials, observers were presented with different target individuals that shared the trait the demonstrator had favoured so that any acquired preference must attach to the trait rather than to a particular individual; or individual: across trials, observers were presented with the same target individuals throughout), type of social stimulus (mating, visual association, acoustic, chemical), type of choice outcome (mating, association time), sex of observer individual (female or male), whether the individual is a virgin (yes or no; if not specified, then it was coded as NA), and demonstrator individual age (same age or different age from the observer). Across original studies, observers' responses were quantified using a variety of measures, including association time with the target stimuli, the number of responses, and the number of mating bouts. Henceforth, we refer to these measures and responses collectively as ‘mate choice’.

Third, we obtained data to calculate effect sizes (e.g. means, s.d.s. or s.e.s., and sample sizes for the control and treatment groups; or the number of individuals who chose each target stimulus) from figures, tables, or texts. Values reported only in figures were extracted with the assistance of a generative artificial intelligence tool and validated against an established digitisation package (see ‘Use of artificial intelligence tools’). If information was ambiguous or missing, the study was excluded from our dataset (see Table S4). Data were primarily extracted by E.S.A.S., with 10% of records checked for accuracy by M.L.

### Effect-size calculation

Following the original meta-analyses of Jones and DuVal (2019) and Davies et al. (2020), we calculated effect-size estimates as Hedges' *g* and OR, so we could investigate the effect of additional effect sizes on either one of the original meta-analyses. When studies reported means, s.d.s, and sample sizes for the treatment and control groups, we used standard formulas to estimate the standardised mean difference as Hedges' *g* (a bias-corrected version of Cohen's *d*). For studies that reported their results as count data, i.e. the number of observer individuals who preferred each stimulus type (control and treatment), we estimated the OR using the standard formula. All formulae and their sampling variances follow the standard meta-analytic treatments of Borenstein et al. (2011). Given that we are updating two meta-analyses that used distinct effect sizes to report their results, to be able to add (combine) our effect sizes to the original datasets, it was necessary to convert the effect sizes from our dataset between the two metrics (Eqns 16–19).

#### Hedges' *g* from group means and s.d.s.

When a study reported the mean, s.d. and sample size of a treatment and a control group, we computed the standardised mean difference *d* using the pooled s.d. *S_p_* (Eqn 1) and applied the small-sample bias correction *J* (Eqn 3) to obtain Hedges' *g* (Eqn 4) and its sampling variance (Eqn 5) (Borenstein et al., 2011):
(1)

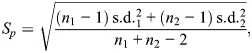

(2)

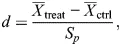

(3)

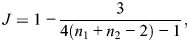

(4)

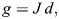

(5)

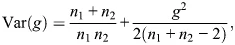
where *n*_1_ and *n*_2_ are the treatment and control sample sizes, respectively. Where only a s.e. was reported, the s.d. was recovered as 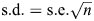.

#### Hedges' *g* from test statistics

For studies reporting a *t*-statistic rather than group descriptives, we converted *t* to *d*, as described by Borenstein et al. (2011), adjusting the degrees of freedom for the experimental design. For a one-sample or paired-sample design (*df*=*n*−1), *d* was obtained from Eqn 6 and its variance from Eqn 9:
(6)

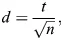

(7)

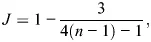

(8)

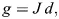

(9)

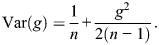


For an independent two-sample design (*df*=*n*_1_+*n*_2_−2), *d* was obtained from Eqn 10:
(10)

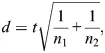

(11)

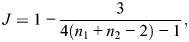

(12)

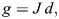

(13)

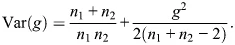


When individual group sizes were not reported for an independent-sample design, we assumed an equal split, *n*_1_=*n*_2_=*N*/2. Effects that were reported only as *F*-statistics, *χ*^2^ values or generalised linear mixed-model outputs were not directly convertible and were therefore excluded from this step.

#### OR from a 2×2 table

For studies reporting binary choice frequencies, we summarised the outcome as an OR [*OR*, which was log-transformed (all mentions of log or ln OR in the manuscript mean the natural logarithm of OR) for model fitting; Eqn 14], with the variance of the log OR was obtained by the delta-method approximation (Eqn 15) (Borenstein et al., 2011):
(14)

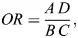

(15)

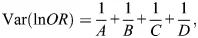
where *A* and *B* are the number of treatment-group individuals choosing the option associated with the social information and the alternative, respectively, and *C* and *D* are the corresponding counts for the control group. When no separate control group existed, we used the null expectation that individuals chose each option with equal probability, setting *C*=*D*=*n*/2, as the two original meta-analyses did. This applied to 22 of the 38 binary effect sizes in our new extraction (11 of 17 studies). Where *n* was odd, the resulting half-integer cell counts were retained rather than rounded because ln(*OR*) and its delta-method variance are defined for noninteger counts; this affected nine effect sizes. No 2×2 table contained a zero cell, so no continuity correction was applied.

#### Conversion between effect-size metrics

To place all studies on a common scale, the effects available in only one metric were converted to the other using the logistic-to-normal approximation (Chinn, 2000; Borenstein et al., 2011). A log OR was converted to a standardised mean difference (Eqn 16) and then bias corrected to *g* (Eqn 18):
(16)

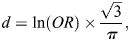

(17)

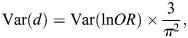

(18)

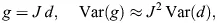
with *J* evaluated as above (*J*≈1, where *n* was unknown). Conversely, Hedges' *g* was converted to a log OR using Eqn 19:
(19)

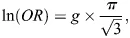

(20)

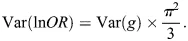


These conversions are approximate and assume that the underlying continuous trait follows a logistic distribution (Chinn, 2000); converted values were flagged in the dataset to distinguish them from directly calculated effects in sensitivity analyses.

### Phylogeny

Our combined sample (original+new datasets) comprised 33 species spanning three broad taxonomic groups: arthropods (e.g. *Drosophila melanogaster*, *Enchenopa binotata*, *Schizocosa ocreata*, *Uca mjoebergi*), fish (e.g. *Poecilia reticulata*, *Gasterosteus aculeatus*, *Oryzias latipes*, *Syngnathus typhle*, *Porichthys notatus*), and other vertebrates including birds (e.g. *Coturnix japonica*, *Taeniopygia guttata*, *Molothrus ater*, *Ficedula hypoleuca*) and mammals (e.g. *Dama dama*, *Mus musculus*, *Rattus norvegicus*). Several families were represented by multiple species (e.g. Poeciliidae: *P. reticulata*, *P. latipinna*, *Poecilia mexicana*, *Poecilia formosa*, *Limia nigrofasciata*, *Limia perugiae*, *Gambusia holbrooki*, *Brachyrhaphis rhabdophora*; Drosophilidae: *D. melanogaster*, *Drosophila subobscura*, *Drosophila simulans*, *Drosophila serrata*; Percidae: *Etheostoma flabellare*, *Etheostoma zonale*, *Etheostoma olmstedi*). We therefore controlled for the non-independence arising from shared evolutionary history by incorporating a phylogeny as a correlation structure in our models (Koricheva et al., 2013).

Because no single published phylogeny included all of our species, we assembled a synthetic tree based on the Open Tree of Life (Hinchliff et al., 2015) using the R package ‘rotl v3.0’ (Michonneau et al., 2016), accessed through ‘prepR4pcm’ (Nakagawa et al., 2026) and following the workflow outlined by Cinar et al. (2022). We retrieved the induced subtree for our species, resolved polytomies at random into a strictly bifurcating topology (‘ape::multi2di’), and, as reliable branch lengths could not be obtained, computed branch lengths using Grafen's (1989) method as implemented in the R package ‘ape v5’ (Paradis et al., 2004). Species names were first reconciled to full binomials and against the Open Tree taxonomy: subspecies were collapsed to the species level (e.g. *Taeniopygia guttata castanotis* became *Taeniopygia guttata*), species recorded only by epithet were resolved to full binomials from their family, and accepted-name synonyms were relabelled to match our dataset (e.g. the fiddler crab returned as *Austruca mjoebergi* was relabelled to *Uca mjoebergi*). After this reconciliation, every species in both datasets was matched to a tip in the Open Tree synthetic tree, so no species required grafting next to a congener.

From the resulting ultrametric tree, we derived a phylogenetic correlation matrix, which we included as a random effect in our multilevel meta-analytic models (see below). We built two trees following identical procedures: one restricted to the 15 species of our own extraction (69 effect sizes) and one for the full combined dataset of 33 species, pooled across our extractions and those of Davies et al. (2020) and Jones and DuVal (2019). The tree for our own extraction (15 species) is shown in Fig. 2, and the tree for the full combined dataset (33 species) in Fig. S1.

### Meta-analysis and meta-regressions

We used the ‘rma.mv’ function from the package ‘metafor v.5.0-1’ (Viechtbauer, 2010) in ‘R v.4.6.0’ (R Core Team, 2025) for our analyses. We started by fitting multilevel mixed-effects meta-analytic models only to the dataset of effect sizes compiled for our study. These meta-analytic models explicitly incorporated random factors: study ID, species ID, and observation ID, which were fitted using the above function (Viechtbauer, 2010). The models also included a variance-covariance matrix to handle dependencies among multiple effect sizes within the same study (assuming *ρ*=0.5 for nested measurements). To account for the non-independence arising from phylogenetic relatedness among species, we included a correlation matrix of phylogenetic relatedness among species as a random effect (Nakagawa and Santos, 2012).

We used the same logic described above to fit separate meta-analytic models to the original OR data of Jones and DuVal (2019) and the Hedges' *d* data of Davies et al. (2020). We combined the Hedges' *d* data of Davies et al. (2020) with our continuous data coded as Hedges' *g*. The last step in this chain was to fit separate meta-analytic models (for Hedges' *g* and for OR) using the combined datasets, thus providing the full updated meta-analytic mean effect of mate choice copying experimental evidence.

We also quantified the total *I*^2^ (a measure of heterogeneity not attributed to sampling error) (Higgins et al., 2003), as well as the proportion of heterogeneity explained by each random factor (partial *I*^2^), which was calculated using the ‘i2_ml’ function from the package ‘orchaRd v.2.2.0’ (Nakagawa et al., 2023b). We created all result plots using the ‘orchard_plot’ and ‘bubble_plot’ functions from the package ‘orchaRd’ (Nakagawa et al., 2023b).

#### Model specification and inference

All models were fitted by restricted maximum likelihood (REML), and inference on fixed effects used *t*-distributed test statistics with containment degrees of freedom (‘test=“t”’, ‘dfs=“contain”’ in ‘rma.mv’), which is preferable to *z*-based inference given the moderate number of clusters available. For every model, we report the variance component of each random effect (*σ*^2^) alongside total and partial *I*^2^ and a 95% prediction interval for the overall mean. We report both because *I*^2^ describes the *proportion* of observed variance not attributable to sampling error, rather than its absolute magnitude, and is sensitive to the precision of the contributing studies (Borenstein et al., 2017; IntHout et al., 2016); the prediction interval expresses the expected range of true effects in a new study on the original scale.

#### Meta-regression models

We fitted the taxonomic group (arthropods, fish, and other vertebrates) as an intercept-coded categorical moderator on each combined dataset, following the heterogeneous-variance workflow of ‘orchaRd 2.0’ (Nakagawa et al., 2023b). Because residual variance differed markedly among taxonomic groups, we allowed the effect-size-level variance to differ by group, specified as a heteroscedastic compound-symmetric structure (‘struct=“HCS”’) on ‘tax_group | identifierEffectSizeID' with the between-group correlation fixed at zero (‘rho=0’), so that one residual variance is estimated per group with no cross-group covariance. A single pooled between-study variance (‘1 | identifierStudyId’) and a homoscedastic species variance (‘1 | taxonomySpecies’) were retained. As in the intercept-only models, a phylogenetic correlation matrix was included as an additional random effect. As in the main analyses, the sampling variance-covariance matrix was constructed with ‘metafor::vcalc’ assuming a within-study correlation (*ρ*) of 0.5, and models were fitted by REML with ‘test=“t”’ and ‘dfs=“contain”’. Both models converged at the first attempt using the ‘nlminb’ optimiser without warnings. We report the omnibus *F*-test of the moderator together with group-specific marginalised means and pairwise contrasts obtained with ‘orchaRd::mod_results’ (via ‘emmeans’), so that inference does not depend on an arbitrary reference level.

Five further moderators extracted during data collection – the sex of the observer (female versus male), the copying mechanism (generalised versus individual), the modality of the social cue (visual, chemical, or acoustic), the mating status of the observer, and the relative age of the demonstrator – were not included in our pre-registered analyses, and several of their levels are sparsely sampled. We therefore fitted them as single-moderator multilevel models on the new extraction (both scales) and, where the coding could be harmonised with an original dataset, on the combined data and treat them all as exploratory throughout. Observer sex harmonises with the ‘Choosing_sex’ variable of Davies et al. (2020) and was therefore also fitted on the combined Hedges' *g* data; it cannot be fitted on the combined OR data because Jones and DuVal (2019) synthesised female mate choice copying only and their rows carry no sex variation. The relative age of the demonstrator showed no variation in our extraction (all ‘same age’) and could not be analysed.

### Small-study effects, time-lag patterns, and sensitivity analyses

We assessed small-study and time-lag effects using three approaches, which were applied to both combined datasets (Hedges' *g* and log OR). First, we visually assessed funnel-plot asymmetry – the expected footprint of selective reporting – by plotting the residuals of a meta-analytic model containing all our random factors against effect-size precision (1/s.e.; funnel plots in the Supplementary Information). Second, as a regression-based test, we fitted a multilevel analogue of Egger's regression, using the s.e. (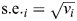) as the precision moderator and, where its slope was significant, refitting with the sampling variance (*v_i_*) as the moderator whose intercept estimates the bias-adjusted mean (Nakagawa et al., 2022; Stanley and Doucouliagos, 2012, 2014); because the sampling variance of standardised mean differences and log ORs depends partly on the estimate itself, we treat this bias-corrected mean as a conservative bound rather than a point estimate (see below). Third, we examined time-lag bias by fitting publication year as a single moderator and then a multi-moderator model containing both publication year and the precision moderator, to test whether either pattern persisted after conditioning on the other.

We estimated the adjusted overall mean using two complementary correction frameworks: the bias-corrected intercept of a multilevel meta-regression on the sampling variance (Nakagawa et al., 2022) and a bias-robust fixed-effect estimator with cluster-robust (CR2) variance estimation (Yang et al., 2024). These two approaches strike different balances between representing the multilevel dependence structure and guarding against selective reporting; we give their full specification and respective limitations below and overlay both corrected means on the orchard plots [‘orchaRd::pub_bias_plot()’].

We tested the robustness of the combined estimates in two further ways, each refitting the model with the same structure as the main analysis. First, leave-one-out and leave-one-species-out analyses removed all effect sizes from one study, or one species, in turn. Second, because the two original meta-analyses overlap in their primary studies but report them on different metrics, we cross-validated by comparing, for every shared study, the Hedges' *g* reported by Davies et al. (2020) with the *g* implied by converting the OR reported by Jones and DuVal (2019) for the same experiment and outcome (Eqn 18); this flagged seven studies whose values disagreed by more than 0.5 Hedges' *g* units. We then refitted each combined model under three versions of the data – the original data (*Main*), a conservative version discarding the seven flagged studies (*Leave-out*), and a best-estimate version with their outcomes re-extracted from the primary sources (*Corrected*). Full specifications of the leave-one-out procedure and the exact re-extraction accounting are given below.

#### Multilevel Egger's regression and the conservative bias-corrected mean

We tested whether study precision moderated the effect size using a two-step procedure (Nakagawa et al., 2022; Stanley and Doucouliagos, 2012, 2014): (1) we fitted a multilevel analogue of Egger's regression using the s.e. as the precision proxy, where *v_i_* represents the sampling variance of the *i*th effect size and 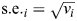; and (2) where the slope of that model was statistically significant – indicating a biased estimate – we refitted the model with the sampling variance (*v_i_*) as the moderator whose intercept provides the bias-adjusted overall meta-analytic mean. We used the s.e. rather than the effective sample size as the precision moderator. For standardised mean differences and log ORs, the sampling variance is partly a function of the effect-size estimate itself, so a regression on 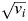 can register asymmetry that is in part an artefact of that dependence (Nakagawa et al., 2022). Because this artefact inflates rather than deflates apparent small-study effects, we read the resulting adjusted mean as a conservative bound on the true average effect rather than as a point estimate – the more pessimistic of our two correction frameworks.

#### Bias-robust mean and its assumptions

The second correction framework follows Yang et al. (2024): a bias-robust weighting scheme combined with cluster-robust (CR2) variance estimation. The weighting scheme is a multivariate fixed-effect generalised least-squares estimator in which the weights derive from the sampling variance-covariance matrix (assuming *ρ*=0.5, as elsewhere); inverse-covariance weighting downweights imprecise studies reporting large effects and prevents a study contributing many correlated effect sizes from accruing weight in proportion to their number, making the resulting mean less susceptible to selective reporting than a random-effects mean. Cluster-robust variance estimation, implemented with ‘clubSandwich’ and clustered by study, then restores valid inference under the non-independence of effect sizes within studies. Because this first-stage model contains no random effects, the CR2 small-sample correction is applicable, which it would not be for a multilevel model with crossed random terms (Williams et al., 2025). It also follows, however, that dependence among effect sizes from the same species – crossed with, rather than nested within, study – is represented by neither the weighting nor the clustering. We therefore read the bias-robust mean as a point estimate that is robust to selective reporting but whose interval may be optimistic where species contribute effect sizes across several studies; our multilevel models, which explicitly represent species and phylogeny, provide complementary uncorrected and bias-corrected estimates. Both corrected means are overlaid on the orchard plots using ‘orchaRd::pub_bias_plot()’.

#### Leave-one-out analyses

We assessed the influence of individual studies and species by repeatedly refitting the combined multilevel model, each time removing all effect sizes either from a single study (leave one study out) or from a single species (leave one species out), and comparing each re-estimated pooled mean with the full-data estimate. Every refit used the same model structure as the main analysis: the sampling variance-covariance matrix was recomputed for the reduced dataset (assuming a within-study correlation of *ρ*=0.5), and the phylogenetic random effect was retained, with the species correlation matrix subset to the species that remained. We ran both analyses for the continuous (Hedges' *g*) and binary (log OR) syntheses using the ‘leave_one_out’ and ‘orchard_leave1out’ functions in the ‘orchaRd’ package (Nakagawa et al., 2023b).

#### Cross-dataset validation and the contentious effect sizes

For every study represented in both source datasets, we compared the Hedges' *g* reported by Davies et al. (2020) with the *g* implied by converting the OR reported by Jones and DuVal (2019) for the same experiment and outcome (Eqn 18). This identified seven studies whose two values disagreed by more than 0.5 Hedges' *g* units. We refitted each combined model under three versions of the data, all using the same multilevel structure as the main analysis: the original data as analysed (*Main*), a conservative version that discarded all effect sizes from the seven flagged studies (*Leave-out*), and a best-estimate version that retained every flagged study but substituted outcomes re-extracted from the original sources (*Corrected*). The effect-size count changes slightly relative to *Main* because the correction was not a one-to-one value swap: three overcounted binary (OR) rows were removed, and on the continuous side, one effect size that had been pooled was kept as the original two outcomes reported, while another's non-target effects were not reinstated.

### Use of artificial intelligence tools

Generative artificial intelligence tools were used at three stages of this work. In every case, their output was checked by the authors, who take full responsibility for the content of this manuscript. First, numerical values reported only in figures were extracted using ‘Google Gemini 3.1 Pro’: a cropped figure and its caption were supplied to the model with a prompt requesting the point estimates and error-bar limits, returned as a Markdown table. Because automated figure extraction can fail silently, E.S.A.S. checked every extracted value against the source figure and independently re-extracted with ‘metaDigitise’ (Pick et al., 2019) a validation subset of 20 records; the two methods agreed closely (mean signed *z*-score=−0.10, 95% CI:−0.27 to 0.06). Second, ‘Claude Sonnet 5’ was used to draft the analytical code and to structure the reproducibility repository. E.S.A.S. reviewed and tested all generated code and publicly archived the complete annotated source (see ‘Data and resource availability’), so that every result reported here can be traced to the code that produced it. Third, ‘Claude Opus 5’ was used to review the written manuscript and suggest edits. All suggestions were evaluated by the authors, and E.S.A.S. and S.N. verified every final wording decision. No artificial intelligence tool was used to search the literature, to screen records for eligibility, or to judge study inclusion. No tool made a final analytical or interpretive decision: model specifications, the treatment of contentious effect sizes, and all conclusions drawn from the results were determined by the authors.

## Supplementary Material



10.1242/biolopen.062878_sup1 Supplementary information
