# Supplementary material for: Mate choice copying in non-human animals: an update of two meta-analyses
Source: Biol Open. 2026 Oct 20;15(10):bio062878. doi: 10.1242/bio.062878 (PMC13730131; doi:10.1242/bio.062878)
Supplement: Supplementary file 1 [file biolopen-15-062878-s1.pdf]

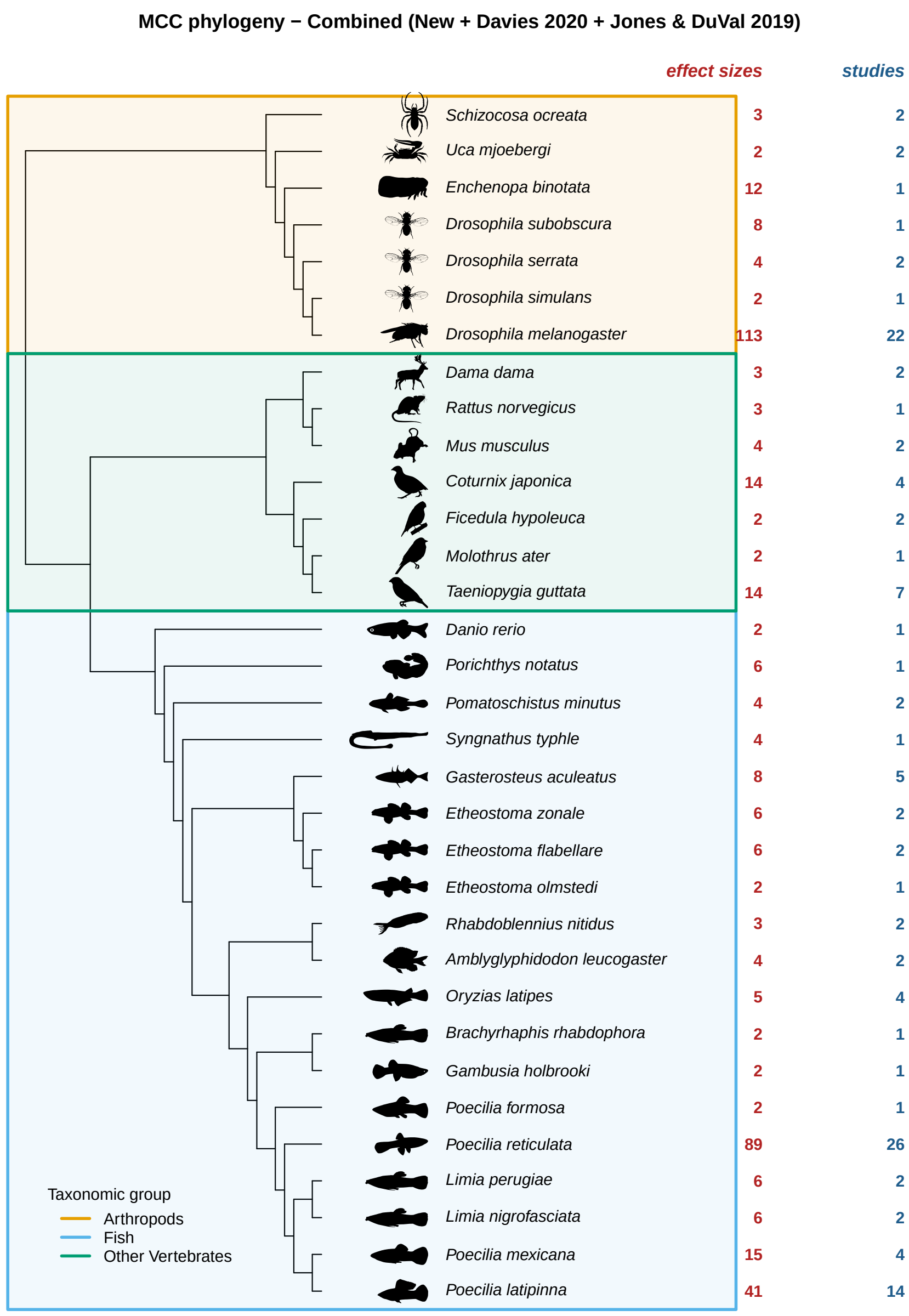

Fig. S1. Phylogenetic tree. The phylogenetic tree of the species included in the meta-analysis using the combined dataset of original and new primary studies, along with the number of effect sizes (k) and the number of studies in which they are reported.

Biology Open • Supplementary information

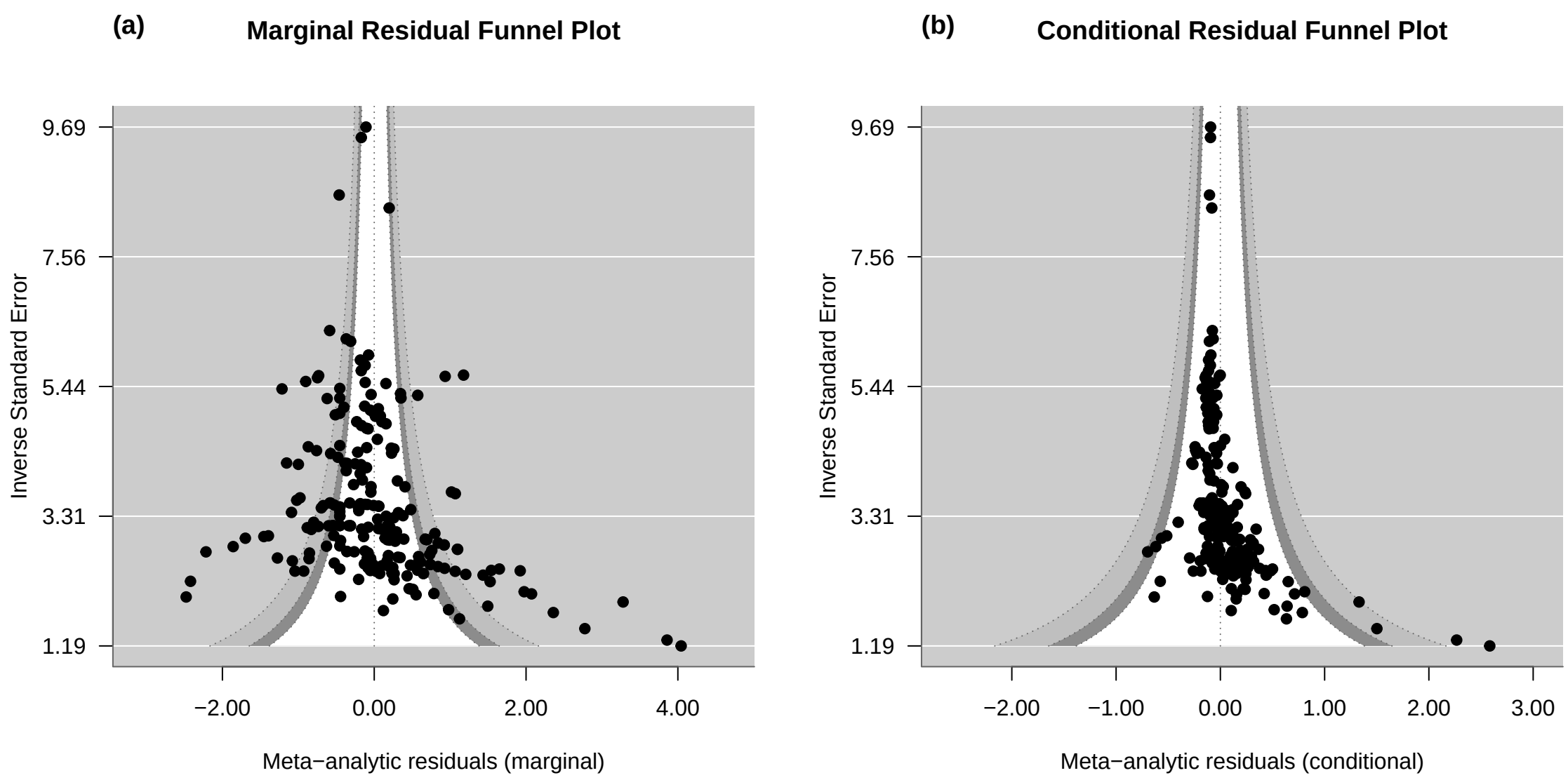

Fig. S2. Funnel plots of Hedges' g data. Both panels plot each effect size against its precision ($1/SE$), but differ in what is removed before plotting: (**a**) Marginal residuals are the deviations of each observed effect from the fixed-effect (intercept, the overall mean) prediction, so they still carry all of the random-effects heterogeneity (between-study, between-effect-size, and between-species–*i.e.* the hierarchical non-independence of effect sizes). Asymmetry here (defined in Methods) is the classic visual signature of small-study and publication bias. (**b**) Conditional residuals additionally subtract the estimated random intercepts (study, effect-size, and species BLUPs), so the heterogeneity captured by the multilevel structure is partialled out. A symmetric, evenly-filled funnel here means that once the multilevel structure is accounted for, there is little precision-related asymmetry left, *i.e.*, the asymmetry seen in (**a**) is largely explained by the modelled random effects rather than by selective reporting. Any asymmetry that persists in (**b**) is the more credible visual indication of true small-study effects.

Biology Open • Supplementary information

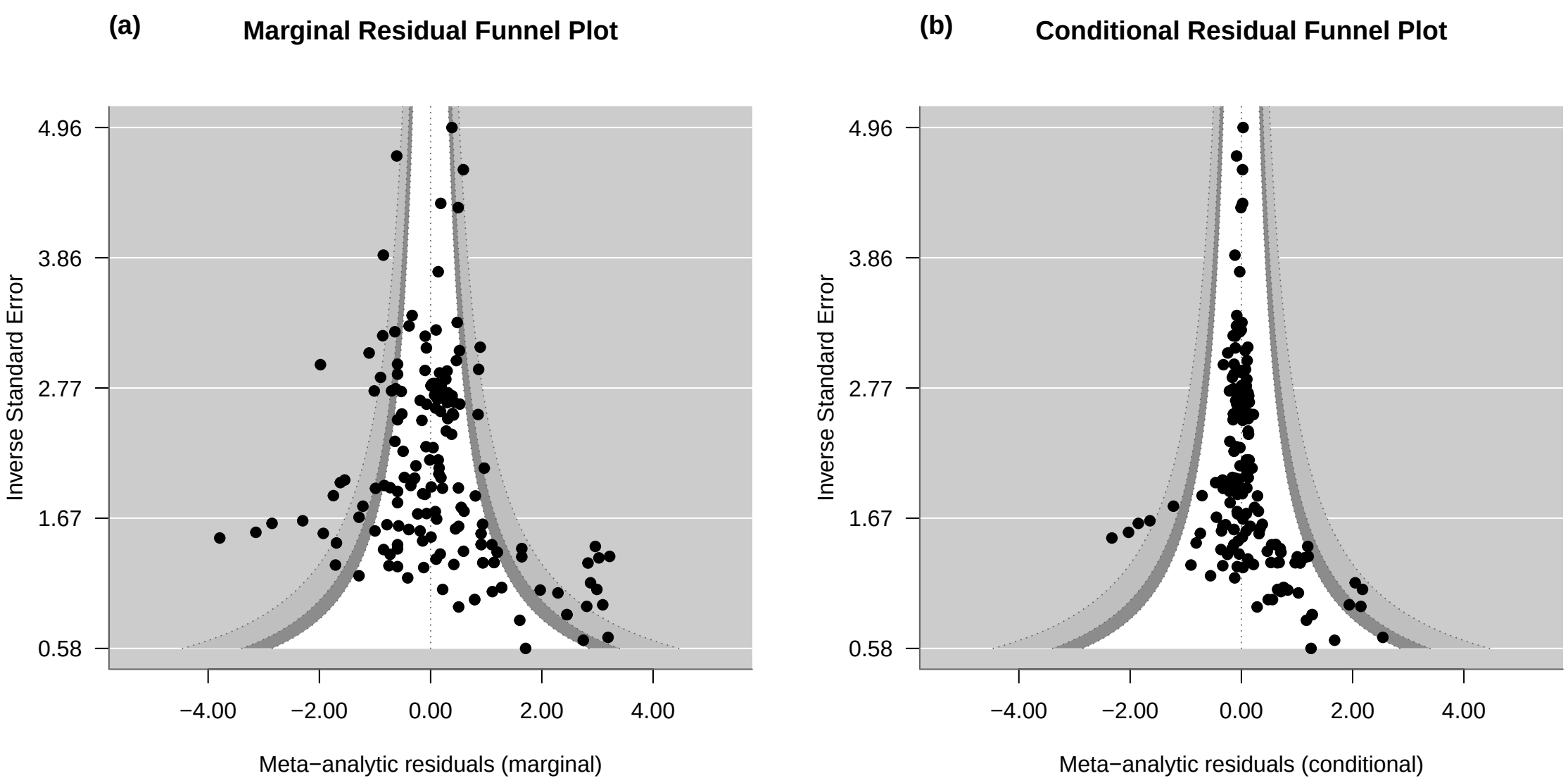

Fig. S3. Funnel plots of odds ratio data. Both panels plot each effect size against its precision ($1/SE$), but differ in what is removed before plotting: (**a**) Marginal residuals are the deviations of each observed effect from the fixed-effect (intercept, the overall mean) prediction, so they still carry all of the random-effects heterogeneity (between-study, between-effect-size, and between-species–*i.e.* the hierarchical non-independence of effect sizes). Asymmetry here (defined in Methods) is the classic visual signature of small-study and publication bias. (**b**) Conditional residuals additionally subtract the estimated random intercepts (study, effect-size, and species BLUPs), so the heterogeneity captured by the multilevel structure is partialled out. A symmetric, evenly-filled funnel here means that once the multilevel structure is accounted for, there is little precision-related asymmetry left, *i.e.*, the asymmetry seen in (**a**) is largely explained by the modelled random effects rather than by selective reporting. Any asymmetry that persists in (**b**) is the more credible visual indication of true small-study effects.

Biology Open • Supplementary information

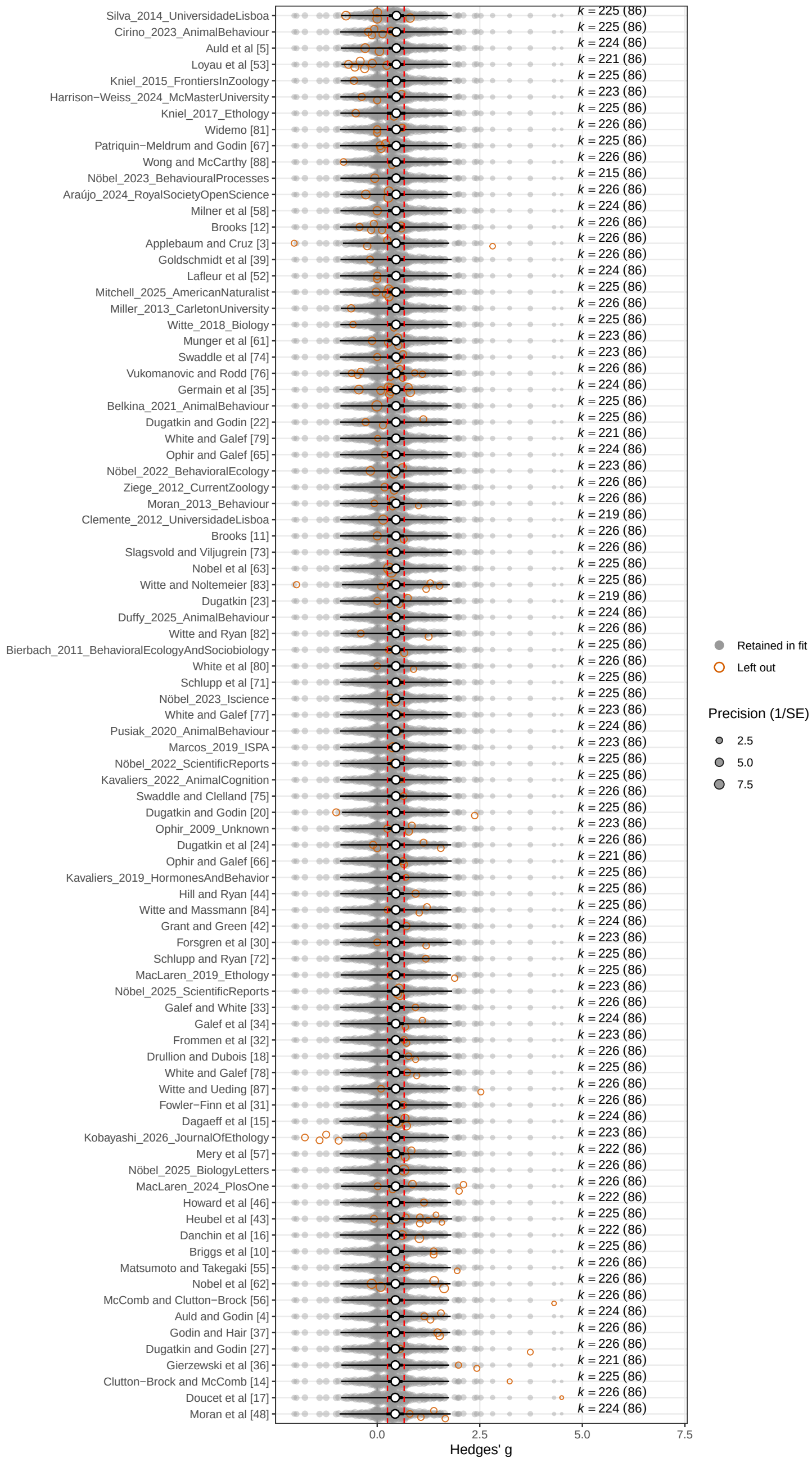

Fig. S4. Leave-one-study-out analysis of the combined Hedges' *g*. Each row re-estimates the multilevel pooled mean after removing one study from the continuous (Hedges' *g*) dataset, with rows ordered by the leave-one-out estimate so the panel reads as an influence ranking. Grey-filled points are the effect sizes retained in that fit, and open orange rings are the effect sizes that were removed, each scaled by its precision ($1/SE$). The white marker is the re-estimated pooled mean, the thick black bar its 95% confidence interval and the thin black whisker its 95% prediction interval. The two vertical dashed red lines mark the full-sample 95% confidence interval, so a mean falling between them indicates that omitting that study leaves the pooled estimate essentially unchanged. Labels on the right give the number of effect sizes retained (k), with the number of studies remaining in parentheses.

Biology Open • Supplementary information

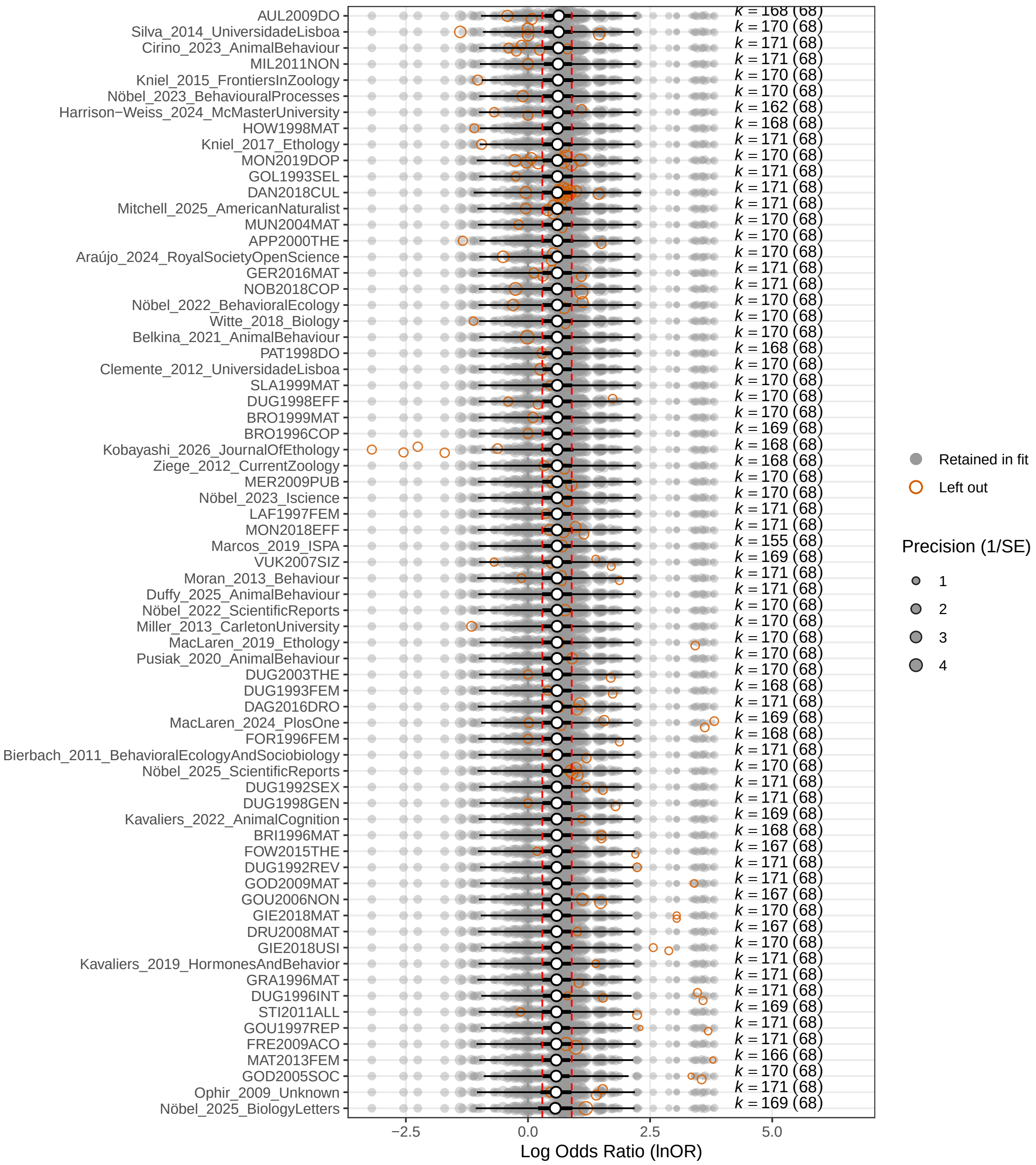

Fig. S5. Leave-one-study-out analysis of the combined odds ratio. Each row re-estimates the multilevel pooled mean (on the log-odds-ratio scale) after removing one study from the binary dataset, with rows ordered by the leave-one-out estimate. Grey-filled points are the effect sizes retained in that fit, and open orange rings are the effect sizes that were removed, each scaled by its precision ($1/SE$). The white marker is the re-estimated pooled mean, the thick black bar its 95% confidence interval, and the thin black whisker its 95% prediction interval. The two vertical dashed red lines mark the full-sample 95% confidence interval, so a mean falling between them indicates that omitting that study leaves the pooled estimate essentially unchanged. Labels on the right give the number of effect sizes retained (k), with the number of studies remaining in parentheses.

Biology Open • Supplementary information

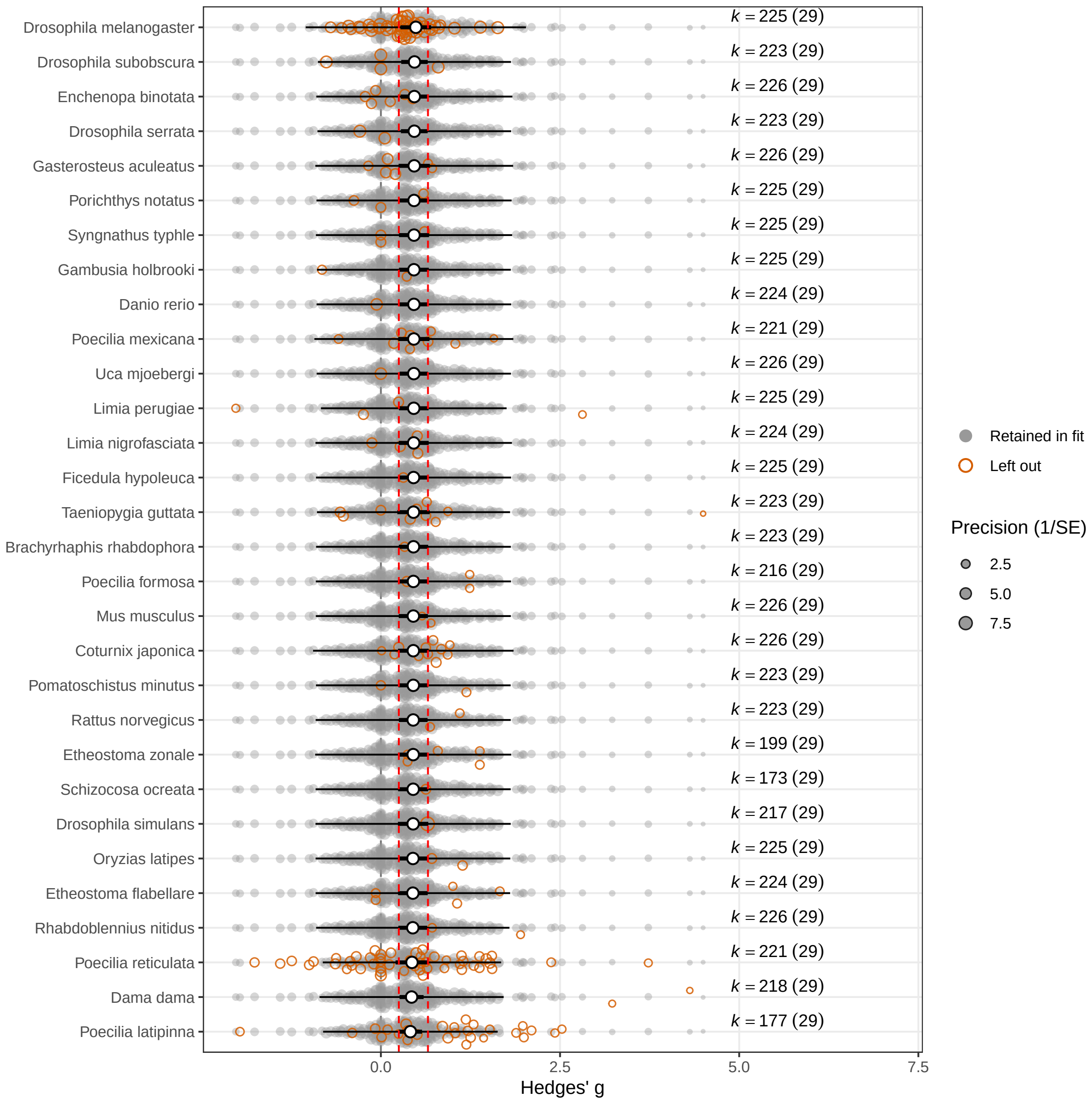

Fig. S6. Leave-one-species-out analysis of the combined Hedges' *g*. Each row re-estimates the multilevel pooled mean after removing all effect sizes from one species in the continuous (Hedges' *g*) dataset—a stricter test than leaving out single studies, because the phylogenetic sampling is uneven. Rows are ordered by the leave-one-out estimate. Grey-filled points are the effect sizes retained in that fit, and open orange rings are the effect sizes contributed by the omitted species, each scaled by its precision ($1/SE$). The white marker is the re-estimated pooled mean, the thick black bar its 95% confidence interval, and the thin black whisker its 95% prediction interval. The two vertical dashed red lines mark the full-sample 95% confidence interval. Labels on the right give the number of effect sizes retained (k), with the number of species remaining in parentheses.

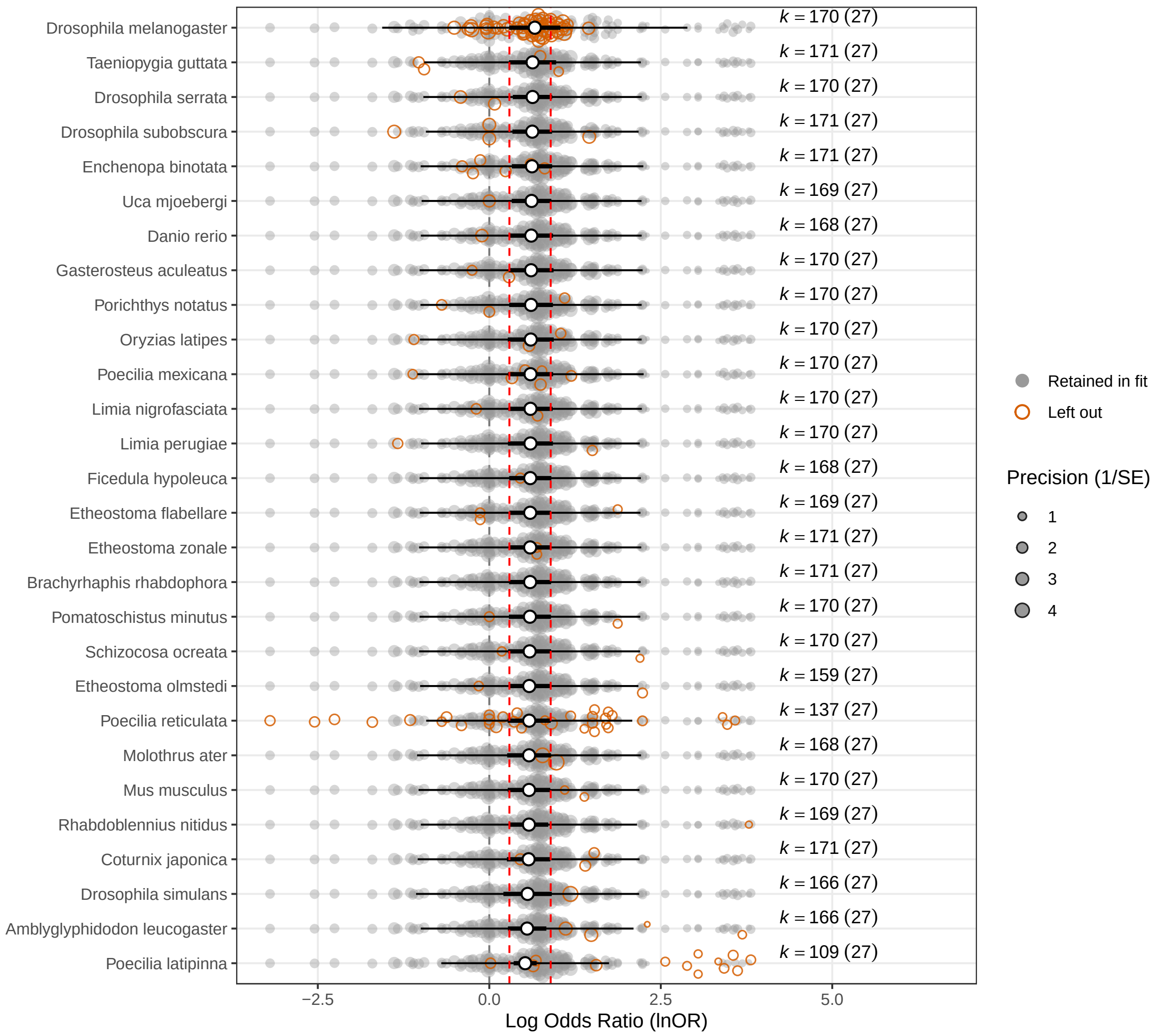

Fig. S7. Leave-one-species-out analysis of the combined odds ratio. Each row re-estimates the multilevel pooled mean (on the log-odds-ratio scale) after removing all effect sizes from one species in the binary dataset—a stricter test than leaving out single studies, because the phylogenetic sampling is uneven. Rows are ordered by the leave-one-out estimate. Grey-filled points are the effect sizes retained in that fit, and open orange rings are the effect sizes contributed by the omitted species, each scaled by its precision ($1/SE$). The white marker is the re-estimated pooled mean, the thick black bar its 95% confidence interval, and the thin black whisker its 95% prediction interval. The two vertical dashed red lines mark the full-sample 95% confidence interval. Labels on the right give the number of effect sizes retained (k), with the number of species remaining in parentheses.

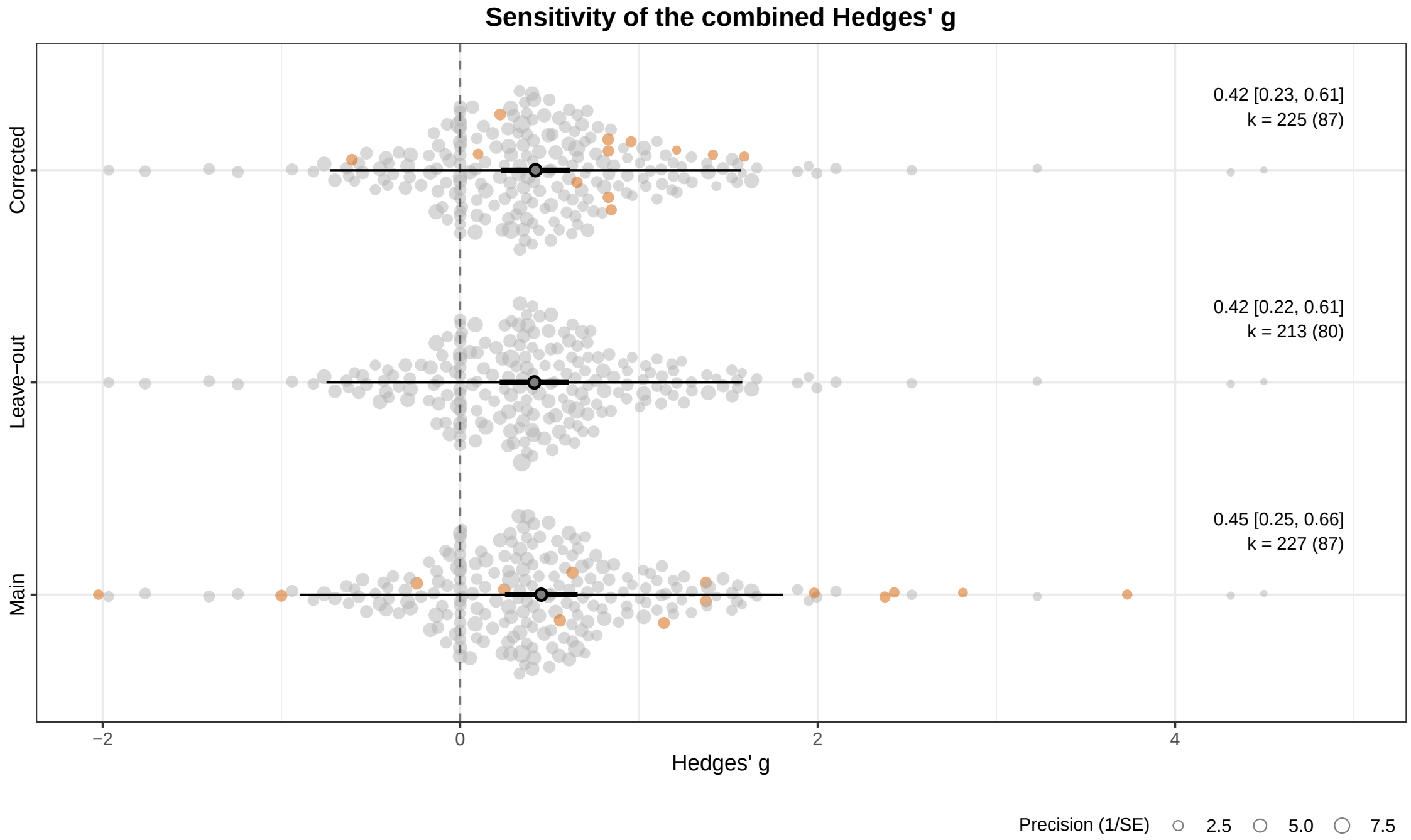

Fig. S8. Sensitivity of the combined Hedges' *g* to the contentious effect sizes. Each row shows the combined random-effects estimate for the continuous (Hedges' *g*) dataset under one version of the data: the original analysis (*Main*), the conservative version with all seven flagged studies removed (*Leave-out*), and the best-estimate version with their effect sizes replaced by re-extractions (*Corrected*). Points are individual effect sizes scaled by their precision ($1/SE$), with the effect sizes from the seven contentious studies highlighted in orange and all others in grey; the central marker is the pooled mean, the thick bar its 95% confidence interval, and the thin whisker its 95% prediction interval. The vertical dashed line marks the no-effect line ($g = 0$). Annotations give the pooled mean [95% CI] and the number of effect sizes (k), with the number of studies in parentheses.

Biology Open • Supplementary information

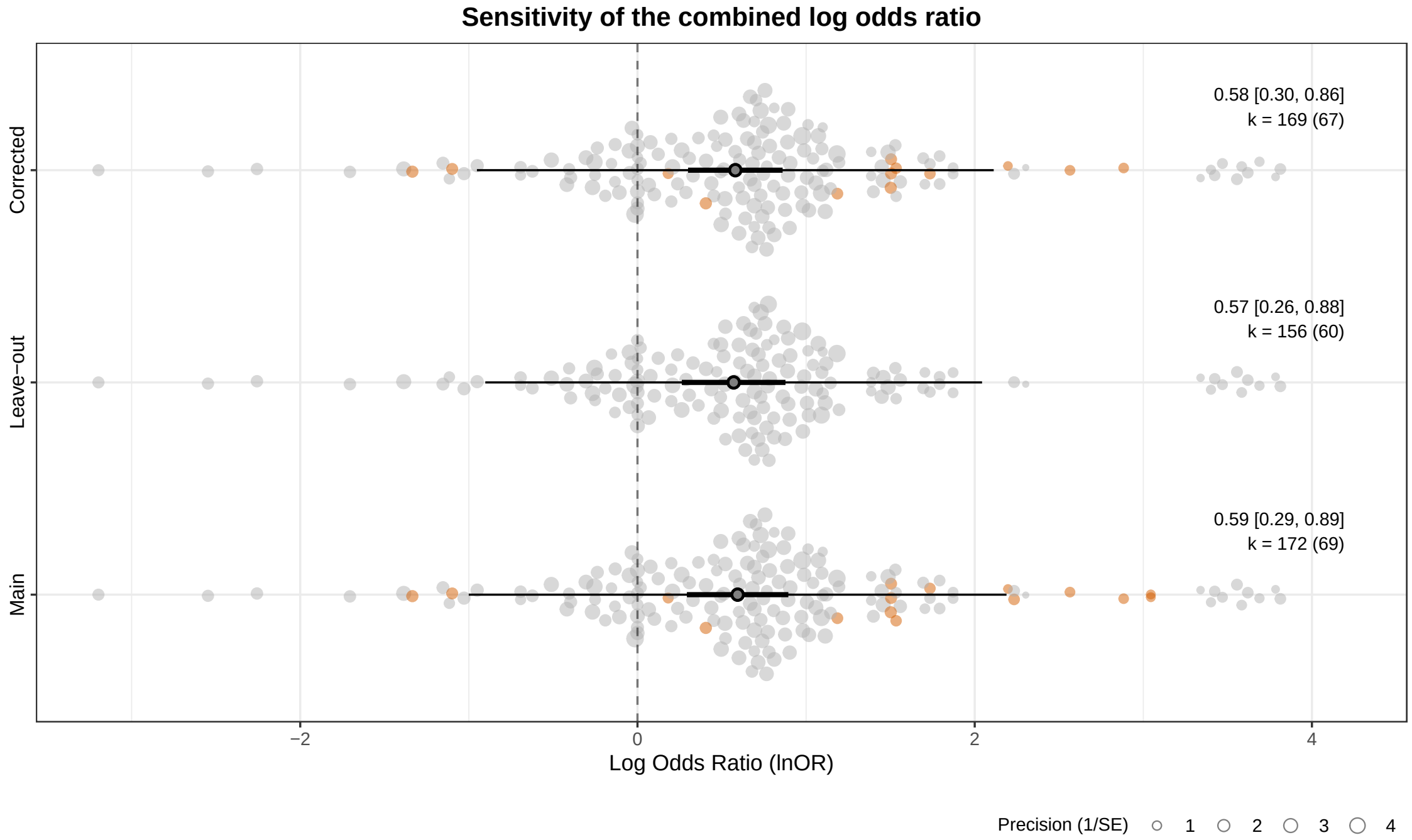

Fig. S9. Sensitivity of the combined odds ratio to the contentious effect sizes. Each row shows the combined random-effects estimate for the binary dataset (on the log-odds-ratio scale) under one version of the data: the original analysis (*Main*), the conservative version with all seven flagged studies removed (*Leave-out*), and the best-estimate version with their effect sizes replaced by re-extractions (*Corrected*). Points are individual log-odds-ratio effect sizes scaled by their precision ($1/SE$), with the effect sizes from the contentious studies highlighted in orange and all others in grey; the central marker is the pooled mean, the thick bar its 95% confidence interval, and the thin whisker its 95% prediction interval. The vertical dashed line marks no effect (lnOR $= 0$, i.e. OR $= 1$). Annotations give the pooled mean [95% CI] and the number of effect sizes (k), with the number of studies in parentheses.

Biology Open • Supplementary information

Table S1. Literature search strings, access dates and records retrieved. All searches covered the full range of available publi-cation dates in every database and search platform (1900 to 2026 inclusive) and were not restricted by language, subject area or doc-ument type. Search strings were adapted to the structure of each database or platform, and translated for searches in non-English languages.

Source	Access date	Query	Records
Web of Science (combined and optimised search terms)	04 February 2026	TS = (("mate choice" AND "copy*") OR ("mate-choice" AND "copy*") OR "mate-copy*" OR "mate copy*" OR "female* copy*")	891
Scopus (Davies et al. search terms)	23 February 2026	TITLE-ABS-KEY("mate choice" AND (copying OR nonindependent OR learning))	718
Scopus (Jones and DuVal search terms)	04 February 2026	TITLE-ABS-KEY (("mate choice" AND "copy*") OR ("mate-choice" AND "copy*") OR "mate-copy*" OR "mate copy*" OR "female* copy*")	474
BASE	05 February 2026	("mate choice" AND "copy*") OR ("mate-choice" AND "copy*") OR "mate-copy*" OR "mate copy*" OR "female* copy*"	212
SciELO	06 February 2026	(title:("mate choice" OR "mate copying" OR "escolha de parceiro" OR "cópia de escolha") OR abstract:("mate choice" OR "mate copying" OR "escolha de parceiro" OR "cópia de escolha") OR subject:("mate choice" OR "mate copying" OR "escolha de parceiro" OR "cópia de escolha"))	11
Google Scholar (Japanese)	26 February 2026	" 配偶者選択" " コピー" OR " 交尾選択" " コピー" OR " 選択のコピー" OR " 配偶者のコピー"	2
Google Scholar (Polish)	26 February 2026	"wybór partnera" "kopiowanie" OR "wybór do rozrodu" "kopiowanie" OR "kopiowanie wyboru" OR "kopiowanie partnera"	77
Google Scholar (Portuguese)	26 February 2026	"escolha de parceiro" "cópia" OR "escolha de acasalamento" "cópia" OR "cópia de escolha" OR "cópia de parceiro"	89

Table S1 continued

Source	Access date	Query	Records
Google Scholar (Russian)	26 February 2026	"выбор партнера" "копирование" OR "выбор для спаривания" "копирование" OR "копирование выбора" OR "копирование партнера"	70
Google Scholar (Simplified Chinese)	26 February 2026	"配偶选择" "复制" OR "交配选择" "复制" OR "选择复制" OR "配偶复制"	93
Google Scholar (Spanish)	26 February 2026	"elección de pareja" "copia" OR "elección de apareamiento" "copia" OR "copia de elección" OR "copia de pareja"	218
**Total**			**2,855**

Table S2. PRISMA-EcoEvo [1] reporting checklist for the present system-atic review and meta-analysis. “Reported” status: Yes = fully reported; Partly = reported but incomplete; No = not reported; NA = not applica-ble. Locations are given as manuscript section headings (and subheadings where available) rather than page numbers, so they remain valid through typesetting and repagination. “Methods” abbreviates *Materials and Meth-ods*; “SI” denotes the supplementary information.

Item	Sub-item	Reported?	Location / notes
**Title and abstract**			
1.1	Identify the review as a systematic review, meta-analysis, or both	Yes	Abstract identifies it as a meta-analysis update
1.2	Summarise the aims and scope of the review	Yes	Abstract
1.3	Describe the data set	Yes	Abstract: 69 new effect sizes from 29 studies combined with original datasets
1.4	State the results of the primary outcome	Yes	Abstract: positive, significant effect on both scales
1.5	State conclusions	Yes	Abstract: effect is modest and context-dependent
1.6	State limitations	Yes	Abstract notes high heterogeneity and small-study effects
**Aims and questions**			
2.1	Provide a rationale for the review	Yes	Introduction
2.2	Reference any previous reviews or meta-analyses on the topic	Yes	Update of Jones & DuVal and Davies et al. (Introduction)
2.3	State the aims and scope of the review (including its generality)	Yes	Introduction / objectives
2.4	State the primary questions the review addresses (e.g. which moderators were tested)	Yes	Two research questions (Introduction); moderators specified (Methods, *Meta-regression models*)
2.5	Describe whether effect sizes were derived from experimental and/or observational comparisons	Yes	PECOS study design: experimental audience-effect designs (Methods, *Eligibility criteria and search protocols*)
**Review registration**			

continued on next page

Biology Open • Supplementary information

Table S2 continued from previous page

Item	Sub-item	Reported?	Location / notes
3.1	Register aims, hypotheses, and methods in a time-stamped, publicly accessible archive and link it in the methods section	Yes	Pre-registered on OSF (Methods (opening); Data availability); protocol cited in reference list and URL given in the methods text
3.2	Describe deviations from the registered aims and methods	Yes	"Additions and deviations" section (Methods, *Additions and deviations*)
3.3	Justify deviations from the registered aims and methods	Yes	Rationales given for each deviation (Methods, *Additions and deviations*)
**Eligibility criteria**			
4.1	Report the specific criteria used for including/excluding studies at title/abstract and full-text screening	Yes	Numbered inclusion criteria + PECOS framework (Methods, *Eligibility criteria and search protocols*)
4.2	Justify criteria, if necessary	Yes	Criteria follow the two source meta-analyses and study scope (Methods, *Eligibility criteria and search protocols*)
**Finding studies**			
5.1	Define the type of search (e.g. comprehensive search, representative sample)	Yes	Comprehensive, multilingual, grey-literature–inclusive search (Methods, *Eligibility criteria and search protocols*)
5.2	State what sources of information were sought	Yes	Scopus, ISI Web of Science, Google Scholar, SciELO, BASE; grey literature (Methods, *Eligibility criteria and search protocols*)
5.3	Include, for each database, the exact search strings used, with keyword combinations and Boolean operators	Partly	Full search strings provided in the SI; databases named in main text (Methods, *Eligibility criteria and search protocols*; Supplementary Table S1)
5.4	Provide enough information to repeat the search, including the timespan covered (start and end dates)	Partly	Publication timespan 1900–2026 stated (Methods, *Eligibility criteria and search protocols*; Supplementary Table S1); exact date each source was last searched not reported
**Study selection**			

continued on next page

Biology Open • Supplementary information

Table S2 continued from previous page

Item	Sub-item	Reported?	Location / notes
6.1	Describe how studies were selected at each stage of screening (e.g. decision trees, software)	Yes	Two-stage screening described (Methods, *Study selection and screening*)
6.2	Report the number of people involved and how they contributed (e.g. independent parallel screening)	Partly	Disagreements resolved by consensus (Methods, *Study selection and screening*); number of screeners and independence not explicitly stated
**Data collection process**			
7.1	Describe where in the reports data were collected from (e.g. text or figures)	Yes	Text and figures (Methods, *Data collection*)
7.2	Describe how data were collected (e.g. software used to digitise figures)	Yes	Data extracted from figures (Google Gemini), validated with metaDigitise (Methods, *Data collection*)
7.3	Describe moderator variables constructed from collected data	Yes	Copying mechanism, cue modality, observer mating status, relative demonstrator age (Methods, *Data collection*)
7.4	Report how missing or ambiguous information was dealt with	Yes	Studies with missing/ambiguous data excluded; effect sizes derived from test statistics (Methods, *Data collection*)
7.5	Report who collected data	Yes	Individuals who performed extraction identified (Methods, *Data collection*)
7.6	State the number of extractions checked for accuracy by co-authors	Yes	Percentage of records checked for accuracy reported (Methods, *Data collection*)
**Data items**			
8.1	Describe the key data sought from each study	Yes	Citation, experiment details, effect-size data (Methods, *Data collection*)
8.2	Describe items that do not appear in main results or could not be extracted	Partly	Non-convertible statistics (F, χ^2, GLMM outputs) excluded (Methods, *Eligibility criteria and search protocols*)
8.3	Describe main assumptions or simplifications made	Yes	Equal-probability assumption ($C = D = n/2$) when no control; outcomes grouped as "mate-choice" (Methods, *Effect size calculation*, *Odds ratio from a* 2×2 *table*)

continued on next page

Biology Open • Supplementary information

Table S2 continued from previous page

Item	Sub-item	Reported?	Location / notes
8.4	Describe the type of replication unit	Yes	Observation-level effect sizes nested within studies/species (Methods, *Model specification and inference*)
**Assessment of individual study quality**			
9.1	Describe whether the quality of included studies was assessed	No	No formal study-level risk-of-bias/quality tool used (publication bias assessed instead)
9.2	Describe how information about study quality was incorporated into analyses	No	Not applicable; study-level quality not assessed
**Effect size measures**			
10.1	Describe effect size(s) used	Yes	Hedges' g and odds ratio (Methods, *Data collection*)
10.2	Provide a reference to the equation of each effect size and its sampling variance	Yes	Equations with citations (Borenstein et al.); delta-method variance (Methods, *Effect size calculation*)
10.3	If no reference exists, derive the equations and state the assumed sampling distribution(s)	Yes	Conversions derived, logistic-distribution assumption stated (Methods, *Conversion between effect-size metrics*)
**Missing data**			
11.1	Describe steps taken to deal with missing data during analysis	Partly	Studies with missing data excluded; converted values flagged for sensitivity analysis (Methods, *Data collection*)
11.2	Justify the decisions made to deal with missing data	Partly	Conversions noted as approximate and checked in sensitivity analyses (Methods, *Conversion between effect-size metrics*)
**Meta-analytic model description**			
12.1	Describe the models used for synthesis of effect sizes	Yes	Multilevel mixed-effects models via `rma.mv` (Methods, *Meta-analysis and meta-regressions*)

continued on next page

Biology Open • Supplementary information

Table S2 continued from previous page

Item	Sub-item	Reported?	Location / notes
12.2	If not a random-effects model, justify the choice	Partly	Multilevel random-effects model used; rationale conveyed implicitly rather than as an explicit justification (Methods, *Meta-analysis and meta-regressions*)
**Software**			
13.1	Describe the statistical platform used for inference	Yes	R (Methods, *Model specification and inference*)
13.2	Describe the packages used to run models	Yes	`metafor`, `orchaRd` (Methods, *Model specification and inference*)
13.3	Describe the functions used to run models	Yes	`rma.mv` (Methods, *Model specification and inference*)
13.4	Describe any arguments that differed from default settings	No	Non-default arguments not described in the main text
13.5	Describe the version numbers of all software used	Yes	R v4.6.0, `metafor` v5.0-1 (Methods, *Model specification and inference*)
**Non-independence**			
14.1	Describe the types of non-independence encountered	Yes	Multiple effect sizes per study, species, and phylogenetic non-independence (Methods, *Model specification and inference*)
14.2	Describe how non-independence has been handled	Yes	Random effects for study/species/observation ID and a phylogenetic correlation matrix (Methods, *Model specification and inference*)
14.3	Justify decisions made	Yes	Model structure described as accounting for each dependency source (Methods, *Model specification and inference*)
**Meta-regression and model selection**			
15.1	Provide a rationale for the inclusion of moderators evaluated in meta-regressions	Yes	Taxonomic group plus four candidate biological moderators (Methods, *Meta-regression models*)
15.2	Justify the number of parameters estimated relative to effect sizes and studies	Partly	Uni-moderator analyses used; explicit parameter-count justification not detailed (Methods, *Meta-regression models*)

continued on next page

Biology Open • Supplementary information

Table S2 continued from previous page

Item	Sub-item	Reported?	Location / notes
15.3	Describe any process of model selection	Partly	Series of uni-moderator models; no formal model-selection procedure (Methods, *Meta-regression models*)
**Publication bias and sensitivity analyses**			
16.1	Describe assessments of risk of bias due to missing results	Yes	Funnel-plot asymmetry, multilevel Egger's regression, time-lag test (Methods, *Small-study effects, time-lag patterns...*)
16.2	Describe steps taken to investigate the effects of such biases	Yes	Bias-corrected intercept and bias-robust estimator (Methods, *Multilevel Egger's regression...*; Results, *Small-study effects, time-lag patterns...*)
16.3	Describe any other analyses of robustness of the results	Yes	Leave-one-out (study and species), contentious effect-size handling (Methods, *Leave-one-out analyses*; Results, *Small-study effects, time-lag patterns...*)
**Clarification of post hoc analyses**			
17.1	Acknowledge hypotheses formulated after data analysis	Yes	Four moderators explicitly labelled exploratory/candidate (Methods, *Meta-regression models*)
**Metadata, data, and code**			
18.1	Share metadata (data descriptions)	Yes	GitHub repository and supplementary website (Data availability)
18.2	Share data required to reproduce the results presented	Yes	Data available on GitHub (Data availability)
18.3	Share additional data not presented in the manuscript	Yes	Supplementary materials / website (Data availability)
18.4	Share analysis scripts	Yes	Annotated R code on GitHub / website (Data availability)
**Results of study selection process**			
19.1	Report the number of studies screened	Yes	PRISMA flowchart, Figure 1
19.2	Report the number of studies excluded at each stage of screening	Yes	PRISMA flowchart, Figure 1

continued on next page

Table S2 continued from previous page

Item	Sub-item	Reported?	Location / notes
19.3	Report brief reasons for exclusion from the full-text stage	Yes	Reasons listed (n = 19 excluded) (Figure 1; Supplementary Table S4)
19.4	Present a PRISMA-like flowchart	Yes	Figure 1
**Sample sizes and study characteristics**			
20.1	Report the number of studies and effect sizes for data included in meta-analyses	Yes	69 effect sizes from 29 new studies, combined with source datasets (Results, *Screening outcomes and dataset characteristics*)
20.2	Report the number of studies and effect sizes for subsets included in meta-regressions	Partly	Overall counts reported; per-subset counts for each moderator not fully reported (Results, *Screening outcomes and dataset characteristics*)
20.3	Provide a summary of key characteristics for reported outcomes	Yes	Dataset characteristics and the included-studies table (Results, *Screening outcomes and dataset characteristics*; Supplementary Table S3)
20.4	Provide a summary of limitations of included moderators (e.g. collinearity/overlap)	No	Moderator collinearity/overlap not summarised
20.5	Provide a summary of characteristics related to individual study quality (risk of bias)	No	Not applicable; study-level quality not assessed
**Meta-analysis**			
21.1	Provide a quantitative synthesis with mean effect size and confidence/credible intervals	Yes	Overall OR = 1.81 (95% CI 1.34–2.45) and Hedges' g (Abstract; Results, *What is the overall evidence...*)
**Heterogeneity**			
22.1	Report indicators of heterogeneity (e.g. I^2, τ^2, variance components)	Yes	Total $I^2 > 74\%$ and partial I^2 by random factor (Results, *What is the overall evidence...*; Table 1)
**Meta-regression**			

continued on next page

Biology Open • Supplementary information

Table S2 continued from previous page

**Item**	**Sub-item**	**Reported?**	**Location / notes**
23.1	Provide estimates of meta-regression slopes and confidence/credible intervals	Yes	Uni-moderator estimates reported (Results, *Uni-moderator analyses of taxonomic group*; Results, *Exploratory uni-moderator analyses of biological moderators*)
23.2	Include estimates and intervals for all moderator variables assessed (complete reporting)	Yes	Taxonomic and exploratory moderators reported (Results, *Uni-moderator analyses of taxonomic group*; Results, *Exploratory uni-moderator analyses of biological moderators*)
23.3	Report interactions, if included	NA	No interaction terms included (uni-moderator analyses only)
23.4	Describe outcomes from model selection, if done	NA	No formal model selection performed
**Outcomes of publication bias and sensitivity analyses**			
24.1	Provide results for the assessments of the risks of bias	Yes	Funnel plots and Egger's-type regression results (Results, *Small-study effects, time-lag patterns...*; Supplementary Figs S2–S3)
24.2	Provide results for the robustness of the review's results	Yes	Leave-one-out and contentious-effect analyses confirm robustness (Results, *Small-study effects, time-lag patterns...*)
**Discussion**			
25.1	Summarise the main findings in terms of the magnitude of effect	Yes	Discussion
25.2	Summarise the main findings in terms of the precision of effects	Yes	Discussion of intervals/significance
25.3	Summarise the main findings in terms of their heterogeneity	Yes	Substantial, largely unexplained heterogeneity (Discussion, *Mate choice copying as social learning...*)
25.4	Summarise the main findings in terms of their biological/practical relevance	Yes	Implications for sexual selection discussed (Discussion, *Mate choice copying as social learning...*)

continued on next page

Biology Open • Supplementary information

Table S2 continued from previous page

Item	Sub-item	Reported?	Location / notes
25.5	Compare results with previous reviews on the topic	Yes	Direct comparison with the two source meta-analyses (Discussion, *Mate choice copying as social learning...*)
25.6	Consider limitations and their influence on the generality of conclusions	Partly	Evidence limitations discussed (Discussion, *Limitations, knowledge gaps, and future opportunities*); limitations of the review process itself not explicitly discussed
**Contributions and funding**			
26.1	Provide names, affiliations, and funding sources of all co-authors	Yes	CERC and NSERC grants (RGPIN-2026-05996) (Funding)
26.2	List the contributions of each co-author	Yes	CRediT-style author contributions statement (Contribution)
26.3	Provide contact details for the corresponding author	Yes	Corresponding-author contact located in the title page (title page)
26.4	Disclose any conflicts of interest	Yes	“No competing interests declared” (Competing interests)
**References**			
27.1	Provide a reference list of all studies included in the review	Yes	Included studies cited [42–70] (Results, *Screening outcomes and dataset characteristics*; References)
27.2	List included studies as referenced sources	Yes	Included studies appear in the reference list rather than only in a table (Results, *Screening outcomes and dataset characteristics*; References)

Biology Open • Supplementary information

Table S3. Studies included in the updated meta-analysis.

Author	Year	Title	Journal / Institution	DOI / Link
Araújo et al.	2024	A Social Learning Primacy Trend in Mate-Copying: An Experiment in Drosophila Melanogaster.	Royal Society Open Science	10.1098/rsos.240408
Belkina et al.	2021	Mate Choice Copying in Drosophila Is Probably Less Robust than Previously Suggested.	Animal Behaviour	10.1016/j.anbehav.2021.04.007
Bierbach et al.	2011	Sperm Competition Risk Affects Male Mate Choice Copying.	Behavioral Ecology and Sociobiology	10.1007/s00265-011-1177-3
Cirino et al.	2023	Robust Mate Preferences despite Means and Opportunity for Mate Choice Copying in an Insect.	Animal Behaviour	10.1016/j.anbehav.2023.03.018
Clemente	2012	Cultural Transmission of Sexual Preferences in Drosophila Melanogaster: Testing for the Durability of Socially Learnt Preferences.	Universidade de Lisboa	ProQuest
Duffy et al.	2025	Mate Choice Copying Behaviour in the Livebearing Fish Brachyrhaphis.	Animal Behaviour	10.1016/j.anbehav.2025.123148
Harrison-Weiss	2024	Female Choice and Social Decision-Making in a Marine Toadfish (Porichthys Notatus).	Thesis	hdl
Kavaliers et al.	2022	Odor-Based Mate Choice Copying in Deer Mice Is Not Affected by Familiarity or Kinship.	Animal Cognition	10.1007/s10071-021-01550-z
Kavaliers et al.	2019	Conspecific Infection Threat Rapidly Biases the Social Responses of Female Mice: Involvement of Oxytocin.	Hormones and Behavior	10.1016/j.yhbeh.2019.04.016
Kniel et al.	2017	The Role of the Model in Mate-Choice Copying in Female Zebra Finches.	Ethology	10.1111/eth.12611
Kniel et al.	2015	Quality of Public Information Matters in Mate-Choice Copying in Female Zebra Finches.	Frontiers in Zoology	10.1186/s12983-015-0119-8
Kobayashi and Karino	2026	Number Matters: Effect of the Number of Models on Mate-Choice Choice Copying in Female Guppies.	Journal of Ethology	10.1007/s10164-025-00876-2

Continued on next page

Table S3 – continued from previous page

Author	Year	Title	Journal / Institution	DOI / Link
MacLaren	2024	Aesthetic Preference for Artificially Selected Color Variant Affects Mate Choice Copying Behavior in Female Poecilia Latipinna.	PLOS One	10.1371/journal.pone.0298171
MacLaren	2019	Evidence of an Emerging Female Preference for an Artificial Male Trait and the Potential for Spread via Mate Choice Copying in Poecilia Latipinna.	Ethology	10.1111/eth.12885
Marcos	2019	Regulação Do Mate-Choice Copying Por Neurónios Apetitivos Ou Neurónios Aversivos de Dopamina Em Drosophila Melanogaster.	ISPA	ProQuest
Miller	2013	Testing for a Link between Personality and Mate-Choice Copying Tendency in the Trinidadian Guppy (Poecilia Reticulata).	Carleton Univesrity	Carleton
Mitchell et al.	2025	Chemical Mate Choice Copying in Drosophila Melanogaster.	American Naturalist	10.1086/736329
Moran et al.	2013	Mate Choice Copying in Two Species of Darters (Percidae: Etheostoma).	Behaviour	10.1163/1568539X-00003092
Nöbel et al.	2025	Long-Term Social Memory of Mate Copying in Drosophila Melanogaster Is Localized in Mushroom Bodies.	Scientific Reports	10.1038/s41598-025-88535-x
Nöbel et al.	2023	Mate Copying Requires the Coincidence Detector Rutabaga in the Mushroom Bodies of Drosophila Melanogaster.	iScience	10.1016/j.isci.2023.107682
Nöbel and Kaufmann	2025	Mate Copying in Drosophila Simulans.	Biology Letters	10.1098/rsbl.2025.0070
Nöbel et al.	2022	Female Fruit Flies Copy the Acceptance, but Not the Rejection, of a Mate.	Behavioral Ecology	10.1093/beheco/arac071
Nöbel et al.	2022	2-D Sex Images Elicit Mate Copying in Fruit Flies.	Scientific Reports	10.1038/s41598-022-26252-5
Nöbel et al.	2023	No Evidence for Mate Copying in Danio Rerio.	Behavioural Processes	10.1016/j.beproc.2023.104837
Ophir	2009	Mate Assessment and Non-Independent Mate Choice by Female Japanese Quail.	McMaster University	hdl

Continued on next page

Biology Open • Supplementary information

Table S3 – continued from previous page

Author	Year	Title	Journal / Institution	DOI / Link
Pusiak et al.	2020	Relative Sexual Attractiveness Does Not Influence Mate-Choice Copying in Male Trinidadian Guppies, Poecilia Reticulata.	Animal Behaviour	10.1016/j.anbehav.2020.02.008
Silva	2014	Can Social Learning Promote Hybridisation? : Mate-Choice Cpying in Drosophila Subobscura Populations.	Universidade de Lisboa	hdl
Witte et al.	2018	Test of the Deception Hypothesis in Atlantic Mollies Poecilia Mexicana—Does the Audience Copy a Pretended Mate Choice of Others?	Biology	10.3390/biology7030040
Ziege et al.	2012	A Comparison of Two Methods to Assess Audience-Induced Changes in Male Mate Choice.	Current Zoology	10.1093/czoolo/58.1.84

Biology Open • Supplementary information

Table S4. Studies excluded from the meta-analysis after full-text assessment, with reasons for exclusion.

Author	Year	Title	Journal	DOI / Source	Reason for exclusion
Bridges et al.	2019	Animal Behaviour: Conformity and the Beginnings of Culture in an Insect.	Current Biology	10.1016/j.cub.2019.01.023	Not the type of study necessary
Chen et al.	2021	Love the Love of Other Men: Male's Mate-Choice Copying under the Activation of Mating Motives; 君子不夺人所好？择偶动机启动下的男性择偶复制	Journal of Psychological Science	10.16719/j.cnki.1671-6981.20210424	Study on humans
Cruz	2014	Communication Networks in the Zebrafish, Danio rerio.	Dissertation Universidade de Lisboa	https://search.proquest.com/openview/aaeafb1c91f2d0dfbd0dbd892c8ce4fe/1?pq-origsite=gscholar&cbl=2026366&diss=y	Not the study design necessary
Durey et al.	2008	Do Female Siamese Fighting Fish Copy the Mate Choice of Others?	European Conference on Behavioural Biology	https://researchprofiles.ku.dk/da/publications/b2bc1be0-ddbe-11dd-b5fc-000ea68e967b	No full-text available
Gilman et al.	2020	Demonstrating Mate Choice Copying in Spiders Requires Further Research	Current Zoology	10.1093/cz/zoz033	Not the type of study necessary
Harrison-Weiss et al.	2025	Assessing the Cues Required for Mate Choice Copying in the Plainfin Midshipman Fish.	Animal Behaviour	10.1016/j.anbehav.2025.123292	Same data as in Harrison-Weiss 2024 Dissertation
Huckvale	2016	An Investigation into the Influence of Hunger-State on the Mate-Choice Copying Phenomenon in Trinidadian Guppies (Poecilia Reticulata).	Thesis	figshare	Does not contain data to calculate effect sizes

Continued on next page

Biology Open • Supplementary information

Table S4 – continued from previous page

Author	Year	Title	Journal	DOI / Source	Reason for exclusion
Imperio et al.	2020	Female Mating Tactics in Lekking Fallow Deer	Scientific Reports	10.1038/s41598-020-58681-5	Not the study design necessary
李卫卫	2017	择偶复制的影响因素及心理机制	心理技术与应用	10.16842/j.cnki.issn2095-5588.2017.03.008	Study on humans
Mendelson et al.	2023	Could Sexual Selection Be Driven by the Mistaken Inferences of Young Females?	PLOS Biology	10.1371/journal.pbio.3002321	Not the type of study necessary
Nöbel et al.	2022	The Importance of Population Heterogeneities in Detecting Social Learning as the Foundation of Animal Cultural Transmission.	Proceedings of the Royal Society B: Biological Sciences	10.1098/rspb.2022.0431	Does not contain data to calculate effect sizes
Plath et al.	2008	Male Fish Deceive Competitors about Mating Preferences.	Current Biology	10.1016/j.cub.2008.06.067	Not the study design necessary
Plath et al.	2009	Audience Effect Alters Male but Not Female Mating Preferences.	Behavioral Ecology and Sociobiology	10.1007/s00265-008-0672-7	Not the study design necessary
Roberts and Gosling	2004	Manipulation of Olfactory Signaling and Mate Choice for Conservation Breeding: A Case Study of Harvest Mice	Conservation Biology	10.1111/j.1523-1739.2004.00514.x	Not the study design necessary
Sapage and Varela	2020	Two Research Avenues for Future Mate-Choice Copying Studies: A Comment on Davies et Al	Behavioral Ecology	10.1093/beheco/araa075	Not the study design necessary
Schütz and Taborsky	2000	Giant Males or Dwarf Females: What Determines the Extreme Sexual Size Dimorphism in Lamprologus Callipterus ?	Journal of Fish Biology	10.1111/j.1095-8649.2000.tb00485.x	Not the study design necessary
Vega-Trejo et al.	2024	Fear of Mating out	Scientific Reports	10.1038/s41598-024-83465-6	Not the study design necessary

Continued on next page

Table S4 – continued from previous page

Author	Year	Title	Journal	DOI / Source	Reason for exclusion
Yang et al.	2025	Evolutionary Divergence of Developmental Plasticity and Learning of Mating Tactics in Trinidadian Guppies.	Journal of Animal Ecology	10.1111/1365-2656.14043	Not the study design necessary
Ziege et al.	2009	Audience Effects in the Atlantic Molly (Poecilia Mexicana)–Prudent Male Mate Choice in Response to Perceived Sperm Competition Risk?	Frontiers in Zoology	10.1186/1742-9994-6-17	Not the study design necessary

Table S5. Coverage of each candidate biological moderator in the new extraction: effect sizes, backing studies and species per level, and the percentage of effect sizes at that level. Identical across the Hedges' g and log odds ratio scales, because every new effect size carries both.

Moderator	Level	k (effect sizes)	Studies	Species	% of ES
Observer sex	Female	61	26	15	88.4
Observer sex	Male	8	5	4	11.6
Copying mechanism	Generalized	37	15	6	53.6
Copying mechanism	Individual	32	14	10	46.4
Social-cue modality	Visual	59	26	13	85.5
Social-cue modality	Chemical	4	3	2	5.8
Social-cue modality	Acoustic	6	1	1	8.7
Demonstration sub-type (visual)	Association	29	14	9	42.0
Demonstration sub-type (visual)	Copulation	29	14	6	42.0
Demonstration sub-type (visual)	(missing)	11	5	4	15.9
Observer virginity	No	21	10	9	30.4
Observer virginity	Yes	48	19	8	69.6
Demonstrator relative age	Same-age	67	28	15	97.1
Demonstrator relative age	(missing)	2	1	1	2.9

Biology Open • Supplementary information

Table S6. Omnibus moderator test and total heterogeneity for ev-ery uni-moderator multilevel model. Because the models are fitted with `test = "t"`, the omnibus test is an F-statistic with contain-ment degrees of freedom rather than a Wald Q_M chi-square. A high total I^2 alongside a non-significant F means the moderator does not explain the heterogeneity. *df* gives the numerator and denominator degrees of freedom. *Structure* is the residual vari-ance structure the fit cascade selected: HCS lets the within-study variance differ by moderator level.

**Analysis**	**k**	**Total I2 (%)**	**F**	**df**	**p**	**Structure**
Observer sex — Hedges' g (new)	69	81.80	1.95	1, 67	0.17	HCS
Observer sex — log OR (new)	69	81.80	1.94	1, 67	0.17	HCS
Mechanism — Hedges' g (new)	69	81.00	0.31	1, 27	0.58	HCS
Mechanism — log OR (new)	69	81.00	0.30	1, 27	0.59	HCS
Modality — Hedges' g (new)	69	81.70	0.18	2, 12	0.84	HCS
Modality — log OR (new)	69	81.70	0.18	2, 12	0.84	HCS
Demonstration sub-type — Hedges' g (new)	58	84.60	1.13	1, 56	0.29	HCS
Demonstration sub-type — log OR (new)	58	84.50	1.13	1, 56	0.29	HCS
Virginity — Hedges' g (new)	69	82.30	1.61	1, 27	0.21	HCS
Virginity — log OR (new)	69	82.30	1.61	1, 27	0.21	HCS
Observer sex — Hedges' g (new + Davies)	227	85.40	0.17	1, 225	0.68	HCS
Mechanism — Hedges' g (new + Davies)	227	85.50	0.05	1, 225	0.82	HCS
Virginity — log OR (new + Jones)	172	75.10	0.13	1, 170	0.72	HCS

Biology Open • Supplementary information

Table S7. Marginalised mean effect for each moderator level, with 95% confidence and 95% prediction intervals, from `orchaRd::mod_results()`. Estimates are on the scale named in the analysis label; log odds ratios are not back-transformed here. Prediction intervals for the models that used the heteroscedastic structure are approximate.

Analysis	Level	Estimate	CI lower	CI upper	PI lower	PI upper
Observer sex — Hedges' g (new)	Female	0.164	-0.102	0.430	-0.957	1.284
Observer sex — Hedges' g (new)	Male	0.512	-0.022	1.046	-0.701	1.724
Observer sex — log OR (new)	Female	0.300	-0.187	0.787	-1.747	2.347
Observer sex — log OR (new)	Male	0.937	-0.041	1.915	-1.279	3.152
Copying mechanism — Hedges' g (new)	Generalized	0.261	-0.053	0.576	-0.820	1.343
Copying mechanism — Hedges' g (new)	Individual	0.165	-0.126	0.455	-0.910	1.240
Copying mechanism — log OR (new)	Generalized	0.477	-0.097	1.051	-1.500	2.453
Copying mechanism — log OR (new)	Individual	0.302	-0.231	0.835	-1.663	2.267
Social-cue modality — Hedges' g (new)	Visual	0.219	-0.051	0.490	-0.914	1.353
Social-cue modality — Hedges' g (new)	Chemical	0.305	-0.140	0.750	-0.882	1.493
Social-cue modality — Hedges' g (new)	Acoustic	0.081	-0.769	0.931	-1.310	1.472
Social-cue modality — log OR (new)	Visual	0.402	-0.094	0.898	-1.669	2.473
Social-cue modality — log OR (new)	Chemical	0.561	-0.257	1.380	-1.610	2.732
Social-cue modality — log OR (new)	Acoustic	0.147	-1.404	1.698	-2.393	2.686
Demonstration sub-type (visual) — Hedges' g (new)	Association	0.108	-0.298	0.514	-1.222	1.438
Demonstration sub-type (visual) — Hedges' g (new)	Copulation	0.362	-0.023	0.746	-0.962	1.686
Demonstration sub-type (visual) — log OR (new)	Association	0.197	-0.544	0.937	-2.227	2.620

Biology Open • Supplementary information

Analysis	Level	Estimate	CI lower	CI upper	PI lower	PI upper
Demonstration sub-type (visual) — log OR (new)	Copulation	0.659	-0.041	1.360	-1.752	3.071
Observer virginity — Hedges' g (new)	No	0.361	-0.001	0.723	-0.795	1.517
Observer virginity — Hedges' g (new)	Yes	0.088	-0.246	0.422	-1.060	1.236
Observer virginity — log OR (new)	No	0.660	-0.003	1.324	-1.452	2.773
Observer virginity — log OR (new)	Yes	0.162	-0.450	0.773	-1.935	2.258
Observer sex — Hedges' g (new + Davies)	Female	0.454	0.293	0.615	-0.898	1.805
Observer sex — Hedges' g (new + Davies)	Male	0.509	0.244	0.774	-0.859	1.877
Copying mechanism — Hedges' g (new + Davies)	Generalized	0.430	0.209	0.650	-0.959	1.818
Copying mechanism — Hedges' g (new + Davies)	Individual	0.458	0.276	0.641	-0.924	1.841
Observer virginity — log OR (new + Jones)	No	0.645	0.265	1.026	-1.017	2.308
Observer virginity — log OR (new + Jones)	Yes	0.559	0.163	0.955	-1.108	2.225

Biology Open • Supplementary information

Table S8. Between-level contrasts: each non-intercept model coef-ficient is the difference between that level and the reference level, on the effect-size scale, with its 95% confidence interval and p-value. A contrast whose interval excludes zero would be statistically distinct; none is.

Analysis	Contrast	Difference	CI lower	CI upper	p
Observer sex — Hedges' g (new)	Male – Female	0.348	-0.150	0.846	0.168
Observer sex — log OR (new)	Male – Female	0.637	-0.275	1.549	0.168
Copying mechanism — Hedges' g (new)	Individual – Generalized	-0.097	-0.454	0.260	0.582
Copying mechanism — log OR (new)	Individual – Generalized	-0.175	-0.827	0.477	0.587
Social-cue modality — Hedges' g (new)	Chemical – Visual	0.086	-0.274	0.446	0.636
Social-cue modality — Hedges' g (new)	Acoustic – Visual	-0.139	-1.031	0.754	0.741
Social-cue modality — log OR (new)	Chemical – Visual	0.159	-0.501	0.820	0.632
Social-cue modality — log OR (new)	Acoustic – Visual	-0.255	-1.884	1.373	0.738
Demonstration sub-type (visual) — Hedges' g (new)	Copulation – Association	0.254	-0.225	0.733	0.293
Demonstration sub-type (visual) — log OR (new)	Copulation – Association	0.463	-0.410	1.336	0.293
Observer virginity — Hedges' g (new)	Yes – No	-0.273	-0.714	0.168	0.215
Observer virginity — log OR (new)	Yes – No	-0.499	-1.305	0.308	0.215
Observer sex — Hedges' g (new + Davies)	Male – Female	0.055	-0.210	0.321	0.683
Copying mechanism — Hedges' g (new + Davies)	Individual – Generalized	0.029	-0.221	0.278	0.820
Observer virginity — log OR (new + Jones)	Yes – No	-0.086	-0.566	0.394	0.723

Biology Open • Supplementary information

Table S9. Study-level crosswalk across the three sources. 94 unique primary studies; **J** = Jones & DuVal (2019), **D** = Davies et al. (2020), **N** = the new extraction. A row listing two sources is one primary study that both hold. Match candidates were generated from author prefix and year (within ±1) and then adjudicated individually; the rejected candidates are in the next table.

Sources	Year	Species	Jones & DuVal (2019)	Davies et al. (2020)	New extraction
JD	2000	perugiae	APP2000THE	Applebaum and Cruz [3]	–
JD	2009	serrata	AUL2009DO	Auld et al [5]	–
JD	1996	reticulata	BRI1996MAT	Briggs et al [10]	–
JD	1996	reticulata	BRO1996COP	Brooks [11]	–
JD	1999	reticulata	BRO1999MAT	Brooks [12]	–
JD	2016	melanogaster	DAG2016DRO	Dagaeff et al [15]	–
JD	2018	melanogaster	DAN2018CUL	Danchin et al [16]	–
JD	2008	guttata	DRU2008MAT	Drullion and Dubois [18]	–
JD	1992	reticulata	DUG1992SEX	Dugatkin and Godin [27]	–
JD	1993	reticulata	DUG1993FEM	Dugatkin and Godin [20]	–
JD	1998	reticulata	DUG1998EFF	Dugatkin and Godin [22]	–
JD	1998	reticulata	DUG1998GEN	Dugatkin [23]	–
JD	2003	reticulata	DUG2003THE	Dugatkin et al [24]	–
JD	1996	minutu, minutus	FOR1996FEM	Forsgren et al [30]	–
JD	2015	ocreata, schizocosa	FOW2015THE	Fowler-Finn et al [31]	–

Sources	Year	Species	Jones & DuVal (2019)	Davies et al. (2020)	New extraction
JD	2016	melanogaster	GER2016MAT	Germain et al [35]	–
JD	2018	latipinna	GIE2018MAT; GIE2018USI	Gierzewski et al [36]	–
JD	2009	reticulata	GOD2009MAT	Godin and Hair [37]	–
JD	1993	aculeatus	GOL1993SEL	Goldschmidt et al [39]	–
JD	1996	latipes	GRA1996MAT	Grant and Green [42]	–
JD	1998	latipes	HOW1998MAT	Howard et al [46]	–
JD	1997	reticulata	LAF1997FEM	Lafleur et al [52]	–
JD	2013	nitidus	MAT2013FEM	Matsumoto and Takegaki [55]	–
JD	2009	melanogaster	MER2009PUB	Mery et al [57]	–
JD	2011	mjoebergi	MIL2011NON	Milner et al [58]	–
JD	2004	nigrofasciata	MUN2004MAT	Munger et al [61]	–
DN	2013	flabellare, zonale	–	Moran et al [48]	Moran_2013_Behaviour
JD	2018	melanogaster	NOB2018COP	Nobel et al [62]	–
DN	2003	japonica	–	Ophir and Galef [65]	Ophir_2009_Unknown
JD	1998	aculeatus	PAT1998DO	Patriquin-Meldrum and Godin [67]	–
JD	1999	hypoleuca	SLA1999MAT	Slagsvold and Viljugrein [73]	–
JD	2007	reticulata	VUK2007SIZ	Vukomanovic and Rodd [76]	–
N	2024	melanogaster	–	–	Araújo_2024_RoyalSocietyOpenScience
D	2015	reticulata	–	Auld and Godin [4]	–
N	2021	melanogaster	–	–	Belkina_2021_AnimalBehaviour
N	2011	mexicana	–	–	Bierbach_2011_BehavioralEcologyAndSociobiology
N	2023	binotata	–	–	Cirino_2023_AnimalBehaviour
N	2012	melanogaster	–	–	Clemente_2012_UniversidadeLisboa

Biology Open • Supplementary information

Sources	Year	Species	Jones & DuVal (2019)	Davies et al. (2020)	New extraction
D	1993	dama	–	Clutton-Brock and McComb [14]	–
J	1992	reticulata	DUG1992REV	–	–
J	1996	reticulata	DUG1996INT	–	–
D	2004	guttata	–	Doucet et al [17]	–
N	2025	rhabdophora	–	–	Duffy_2025_AnimalBehaviour
J	2009	ater	FRE2009ACO	–	–
D	2009	aculeatus	–	Frommen et al [32]	–
J	2005	latipinna	GOD2005SOC	–	–
J	1997	leucogaster	GOU1997REP	–	–
J	2006	leucogaster	GOU2006NON	–	–
D	1998	japonica	–	Galef and White [33]	–
D	2008	norvegicus	–	Galef et al [34]	–
N	2024	notatus	–	–	Harrison-Weiss_2024_McMasterUniversity
D	2008	formosa, latipinna, mexicana	–	Heubel et al [43]	–
D	2006	latipinna	–	Hill and Ryan [44]	–
N	2019	musculus	–	–	Kavaliers_2019_HormonesAndBehavior
N	2022	musculus	–	–	Kavaliers_2022_AnimalCognition
N	2015	castanotis	–	–	Kniel_2015_FrontiersInZoology
N	2017	castanotis	–	–	Kniel_2017_Ethology
N	2026	reticulata	–	–	Kobayashi_2026_JournalOfEthology
D	2012	melanogaster	–	Loyau et al [53]	–
J	2018	melanogaster	MON2018EFF	–	–
J	2019	melanogaster	MON2019DOP	–	–
N	2019	latipinna	–	–	MacLaren_2019_Ethology
N	2024	latipinna	–	–	MacLaren_2024_PlosOne
N	2019	melanogaster	–	–	Marcos_2019_ISPA

Sources	Year	Species	Jones & DuVal (2019)	Davies et al. (2020)	New extraction
D	1994	dama	–	McComb and Clutton-Brock [56]	–
N	2013	reticulata	–	–	Miller_2013_CarletonUniversity
N	2025	melanogaster	–	–	Mitchell_2025_AmericanNaturalist
D	2018	melanogaster	–	Nobel et al [63]	–
N	2022	melanogaster	–	–	Nöbel_2022_BehavioralEcology
N	2022	melanogaster	–	–	Nöbel_2022_ScientificReports
N	2023	rerio	–	–	Nöbel_2023_BehaviouralProcesses
N	2023	melanogaster	–	–	Nöbel_2023_Iscience
N	2025	simulans	–	–	Nöbel_2025_BiologyLetters
N	2025	melanogaster	–	–	Nöbel_2025_ScientificReports
D	2004	japonica	–	Ophir and Galef [66]	–
N	2020	reticulata	–	–	Pusiak_2020_AnimalBehaviour
J	2011	olmstedi	STI2011ALL	–	–
D	1996	latipinna	–	Schlupp and Ryan [72]	–
D	1994	latipinna	–	Schlupp et al [71]	–
N	2014	subobscura	–	–	Silva_2014_UniversidadeLisboa
D	2009	guttata	–	Swaddle and Cleland [75]	–
D	2005	guttata	–	Swaddle et al [74]	–
D	1999	japonica	–	White and Galef [77]	–
D	2000	japonica	–	White and Galef [78]	–
D	2000	japonica	–	White and Galef [79]	–
D	2017	reticulata	–	White et al [80]	–
D	2005	typhle	–	Widemo [81]	–
D	2003	latipinna	–	Witte and Massmann [84]	–

Biology Open • Supplementary information

Sources	Year	Species	Jones & DuVal (2019)	Davies et al. (2020)	New extraction
D	2002	latipinna	–	Witte and Noltemeier [83]	–
D	1998	latipinna	–	Witte and Ryan [82]	–
D	2003	latipinna	–	Witte and Ueding [87]	–
N	2018	mexicana	–	–	Witte_2018_Biology
D	2009	holbrooki	–	Wong and McCarthy [88]	–
N	2012	mexicana	–	–	Ziege_2012_CurrentZoology

Biology Open • Supplementary information

Table S10. Candidate study matches generated by the author-prefix and year rule and rejected on inspection, with the reason. Listing them makes the judgement calls in the unique-study count auditable rather than implicit.

**Candidate pair**	**Why it is not the same study**
DUG1992REV || Dugatkin and Godin [20]	year mismatch (1992 vs 1993)
DUG1992REV || Dugatkin and Godin [27]	Davies [27], labelled 'Dugatkin and Godin 1992', is in fact the single-authored Dugatkin (1992) and pairs with DUG1992SEX; DUG1992REV (Dugatkin and Godin 1992, choice reversal) has no Davies counterpart
DUG1992SEX || Dugatkin and Godin [20]	year mismatch (1992 vs 1993)
DUG1993FEM || Dugatkin and Godin [27]	year mismatch (1993 vs 1992)
DUG1998EFF || Dugatkin [23]	DUG1998EFF pairs with the co-authored Davies [22]; Davies [23] is the single-authored Dugatkin (1998)
DUG1998GEN || Dugatkin and Godin [22]	DUG1998GEN pairs with the single-authored Davies [23]
GIE2018USI || Gierzewski et al [36]	GIE2018USI duplicates GIE2018MAT within Jones & DuVal (the same two reversal-vs-control contrasts under two study codes); folded into GIE2018MAT
NOB2018COP || Nobel et al [63]	NOB2018COP pairs with Davies [62]; Davies [63] is the second Nobel et al. (2018) publication and is present in Davies only
Ophir_2009_Unknown || Ophir and Galef [66]	the new-extraction record is Ophir and Galef (2003) Anim. Behav. 66:369-375 (DOI 10.1006/anbe.2003.2229; its n = 15 matches Davies [65] exactly). Davies [66] is their separate 2004 paper

Supplementary references

1. O'Dea, R. E. *et al*. Preferred Reporting Items for Systematic Reviews and Meta-Analyses in Ecology and Evolutionary Biology: A PRISMA Extension. *Biological Reviews* **96,** 1695–1722. doi:10.1111/brv.12721 (2021).
