# Supplementary material for: Mate choice copying in non-human animals: an update of two meta-analyses
Source: Biol Open. 2026 Oct 20;15(10):bio062878. doi: 10.1242/bio.062878 (PMC13730131; doi:10.1242/bio.062878)
Supplement: Supplementary file 2 [file biolopen-15-062878_review_history.pdf]

Mate choice copying in non-human animals: an update of two meta-analyses

Eduardo S. A. Santos, Aleksandra Milenović, Aneta Arct, Anna Lenz, Ayumi Mizuno, Cassidy Schneider, Christine Sosiak, Erick Lundgren, Hao Qin, Iwo Gross, Jimuel Celeste, Kyle Morrison, Mahi Zakir, Marija Purgar, Santiago Ortega, Sergio Hernandez, Shinichi Nakagawa and Malgorzata Lagisz
10.1242/bio.062878

Editor: Daniel Gorelick

Review timeline

Original submission:	14 August 2026
Editorial decision:	24 August 2026
First revision received:	4 September 2026
Accepted:	17 September 2026

Original submission

First decision letter

MS ID#: bio.062878

MS Title: Mate choice copying in non-human animals: an update of two meta-analyses

Authors: Eduardo S. A. Santos; Aleksandra Milenović; Aneta Arct; Anna Lenz; Ayumi Mizuno; Cassidy Schneider; Christine Sosiak; Erick Lundgren; Hao Qin; Iwo Gross; Jimuel Celeste; Kyle Morrison; Mahi Zakir; Marija Purgar; Santiago Ortega; Sergio Hernandez; Shinichi Nakagawa; Malgorzata Lagisz

I have now reached a decision on the above manuscript.

The reviewer reports are shown at the bottom of this email.

As you will see, the reviewers gave favourable reports, but raised some critical points that will require amendments to your manuscript. I hope that you will be able to carry these out, because we would like to be able to accept your paper.

The reviewer reports speak for themselves, and so should be straightforward to address. I should be grateful if you would provide a point-by-point response detailing how you have dealt with the points raised by the reviewers in the 'Response to Reviewers' box. Please attend to all of the reviewers' comments. If you do not agree with any of their criticisms or suggestions please explain clearly why this is so. Note that reviewer #2, minor issues #1: how you address this comment I leave to your discretion (but please provide a justification if you think that the existing flow of the manuscript is fine).

Reviewer 1

Comments for the author

© 2026. Published by The Company of Biologists under the terms of the Creative Commons Attribution License (https://creativecommons.org/licenses/by/4.0/).

This is an extremely comprehensive and impressive study that, in my opinion, simultaneously demonstrates the value of preregistration, replication, quantitative research synthesis, multilingual literature searching, data and code availability, accounting for publication bias, and more. If all meta-analyses were carried out with this level of comprehensiveness and robustness, the overall scientific evidence base would be a lot healthier.

The manuscript reports on a project that updated two influential meta-analyses with a considerably broader multilingual and grey-literature search. The central finding of the project is that the apparent average mate-choice-copying effect survives the addition of newly identified evidence but becomes substantially smaller, highly heterogeneous, and very sensitive to assumptions about small-study effects. This is not necessarily the most novel or impactful finding but it is nevertheless very important and healthy for science to perform such self-checking exercises when new data, tools, and approaches become available. I also note that Biology Open does not use perceived impact as a rejection criterion, and that the content overlap with the recent Oikos paper by Hanna and co ('Male mate-choice copying: a neglected aspect of sexual selection') is not problematic for me because that paper was a literature review while this is a meta-analysis. Even with all of those positives, I do have some suggested minor revisions that I recommend incorporating to make the paper even stronger.

General feedback:

1. Jones and DuVal studied female mate-choice copying only, whereas the updated dataset includes both male and female observers. Therefore, comparing the updated combined odds ratio directly with Jones and DuVal's OR of 2.71 is not completely like-for-like. The simplest fix would be to provide a female-only sensitivity analysis for the odds-ratio dataset, or at minimum test observer sex as a moderator and make the difference in scope explicit.

2. The manuscript states that the project tested whether the 'main findings' of the two previous meta-analyses still hold, but it does not actually retest several of those findings. E.g. Davies et al. found that experimental design significantly affected copying strength, with stronger effects in before-and-after designs than in no-pretest designs. Separately, Jones and DuVal found significant effects of testing conditions and whether social information favoured a relatively less attractive male. Because of this, I would change the wording of the research question(s) a bit to something like 'whether the overall average effect remains supported'. The alternative, which I'm not necessarily suggesting, is that the authors directly retest the main original findings wherever possible.

3. The PRISMA diagram reports 40 Jones and DuVal studies + 58 Davies studies + 29 new studies, equalling 127 total studies, but (if I'm understanding things correctly) the two original datasets overlap. Doesn't this mean that 127 is not actually the number of unique primary studies?

Line-by-line comments:

Page 2, lines 8-9: 'moderately and positively biases mate choice' is a bit awkward. Maybe change to 'has a moderate positive effect on mate choice'.

Page 2, line 13: There is inconsistent UK and US spelling throughout the manuscript, e.g., 'Re-analysed' here but 'reanalyzed' on page 4, line 66. Please use one consistently throughout.

Page 4, line 75: Change 'in Open Science Framework' to 'on the Open Science Framework'.

Page 5, line 90: 'rather than entirely new synthesis' should be 'rather than an entirely new synthesis'.

Page 6, lines 136-138: The sentence beginning 'We did not limit the number of records...' is a bit difficult to follow. Perhaps rephrase as 'We did not limit the number of records returned by the Google Scholar searches; the records were sorted by relevance.' or something like that.

Page 7, lines 149-150: 'different target individuals that maintained a certain trait' is unclear. Perhaps use 'different target individuals that shared the same trait'.

© 2026. Published by The Company of Biologists under the terms of the Creative Commons Attribution License (https://creativecommons.org/licenses/by/4.0/).

Page 11, lines 245-246: This sentence makes it unclear which phylogeny is shown in which figure. Perhaps state explicitly that Figure 2 shows the new extraction and Supplementary Fig. S1 shows the combined dataset.

Page 12, line 253: The within-study correlation is written as 'r = 0.5' here but 'ρ = 0.5' later. Please use the same notation throughout.

Page 18, line 427: p = 0.72 appears twice in the same parentheses.

Page 20, lines 493-495: This seems to be inconsistent with the Methods. The manuscript says here that the coding could not capture before-and-after versus no-pretest designs and association time versus copulation, but experimental design and choice outcome (mating versus association time) are both described as having been extracted on page 7. Please clarify.

Page 22, line 544: 'The honest interpretation is...' sounds unnecessarily subjective. Maybe use 'A cautious interpretation is...' or just 'These results suggest that...'.

Page 22, line 550: Add a comma after 'ways': 'primary studies quantify copying in incompatible ways, forcing approximate conversions...'.

Reviewer 2

Comments for the author

The article titled "Mate choice copying in non-human animals: an update of two meta-analyses" updates two previous meta-analyses on mate choice copying in non-human animals by incorporating newly identified studies, harmonizing the two source effect-size metrics, and reanalysing the expanded datasets using phylogenetically informed multilevel models. I believe it is an important work, as the creation and expansion of meta-analyses greatly contribute to the timely understanding of science as a whole, but especially in fast-changing fields such as animal behaviour and cognition research. The article is overall clear and extremely well written, and I especially appreciate the authors' careful methodological descriptions, such as those concerning the meta-analytic models, effect-size calculations/ conversions, and the use of phylogenetic and AI tools. Finally, I also appreciate the steps taken by the authors towards reproducibility and open science, namely preregistration, code availability, and extensive supplementary information. I believe the current version of the article is already very close to being suitable for publication, and I have only a few suggestions that I think could further improve its clarity, transparency, and readability.

Major issues

1. Preregistration protocol

- The preregistration protocol could not be retrieved. When accessing the OSF link provided by the authors, I was not able to access the actual preregistration protocol (i.e., the file named "MateChoiceCopyingMetaAnalysisUpdateProtocol.pdf") due to an error. This might be an error on my end, or even an issue with OSF hosting; however, as the authors refer to the protocol quite often throughout the manuscript, I would recommend double-checking whether the protocol is correctly available. If allowed by the journal, I would also suggest providing the protocol as a supplementary file and/ or adding it to the files hosted on GitHub or Zenodo.

2. PRISMA flowchart/data collection

- It is unclear to me exactly which databases were used for the search for new studies, and how many records were obtained from each one. The authors state, "We conducted a literature search across multiple databases and platforms, including Scopus, ISI Web of Science, Google Scholar (for non-English studies), Scientific Electronic Library Online (SciELO), and Bielefeld Academic Search Engine"; however, the phrasing (i.e., "including X, Y, Z") makes the reader wonder whether other

© 2026. Published by The Company of Biologists under the terms of the Creative Commons Attribution License (https://creativecommons.org/licenses/by/4.0/).

databases or platforms not described here were also used. Moreover, it is not uncommon to describe in the PRISMA flowchart the number of records obtained from each database. I would recommend that the authors provide this information to improve clarity.

- The PRISMA flowchart states that 898 records retrieved by Jones & DuVal and Davies et al. before 2019 were excluded. This appears to imply that the authors had access to all records retrieved and screened in those meta-analyses, as well as sufficient information to apply comparable inclusion and exclusion criteria to those records. The authors do state that "In this meta-analysis, we used the datasets from Jones and DuVal and Davies et al. as our starting point," and that "We used the PECOS framework to define a single set of eligibility criteria, applied to both the literature search and the two screening stages, following our pre-registered protocol [13] and the criteria of Jones and DuVal and Davies et al." However, it is still not entirely clear how all 898 records were confidently excluded based on the information available from Jones and DuVal and Davies et al. I suggest that the authors clarify how these records were obtained, assessed, and excluded, and whether they were re-screened directly or their exclusion was inferred from the previous meta-analyses.

- The PRISMA flowchart indicates that the authors retrieved 40 studies from Jones and DuVal and 58 studies from Davies et al., which were added to 29 new studies to ultimately obtain a total of 127 studies included in the current manuscript. This appears to imply that the studies retrieved from each previous meta-analysis were unique and that none overlapped between them. However, this is difficult to reconcile with the authors' statement that "...because the two original meta-analyses overlap in their primary studies but report them on different metrics, we cross-validated by comparing, for every shared study, the Hedges' g reported by Davies et al. with the g implied by converting the odds ratio reported by Jones and DuVal for the same experiment and outcome." The relationship between these numbers and the overlapping studies should therefore be clarified, particularly in the PRISMA flowchart and the Results section.

- The Discussion section states: "By updating the datasets from the two original meta-analyses and reanalysing them within a unified, phylogenetically-informed multilevel framework—227 Hedges' g effect sizes from 87 studies and 172 log odds ratios from 69 studies, spanning 33 species—we can now answer the two questions we set out with." These numbers (i.e., the total numbers of effect sizes, studies, log odds ratios, and species) should also be described in the Results, under "Screening outcomes and dataset characteristics," rather than appearing only in the Discussion.

3. Uni-moderator analyses of taxonomic group

- I believe this analysis is very interesting, and these results add important insights to the evolutionary discussion. Was this analysis also performed by Jones & DuVal and/ or Davies et al., or is it original to the current manuscript? If the former, I suggest describing the previous findings, as none are currently provided, and providing a comparative discussion of them. If the latter, I would suggest that the authors highlight the originality of these findings more clearly, both in the Abstract and in the Discussion.

4. Uni-moderator analyses of biological moderators

- A few biological moderators (such as male vs. female and virgin vs. non-virgin animals) are mentioned extensively throughout the manuscript and discussed in comparison with the findings of Jones & DuVal and Davies et al.; however, their numerical results are only described in the text. I would recommend that the authors provide a figure or table to more directly illustrate these findings and facilitate comparison among moderator categories.

Minor issues

1. The article is somewhat long and contains several figures. I suggest that the authors consider whether some of them could be combined into single multi-panel figures (e.g., Figures 3 and 4, or Figures 6 and 7). This could improve the flow of the manuscript without reducing the amount of information presented.

© 2026. Published by The Company of Biologists under the terms of the Creative Commons Attribution License (https://creativecommons.org/licenses/by/4.0/).

2. The findings from the assessments of small-study and publication biases, as well as time-lag patterns and sensitivity analyses, could be better described in the Results section of the main manuscript, rather than primarily in the Supplementary Material, and further explored in the Discussion. Currently, they read mostly as methodological correction or robustness tools.

Reviewer's Responses to Questions

Experimental quality

Are experiments performed using appropriate methods that will answer the question (or test the hypothesis or support the observations) posed by the authors? Is the right tool used for the job?
If 'No', please indicate reasons in Comments for Author box below.
Reviewer #1:
- Yes

Reviewer #2:
- Yes

Were the data analyzed using appropriate statistical tests?
If 'No', please indicate reasons in Comments for Author box below.
Reviewer #1:
- Yes

Reviewer #2:
- Yes

Reproducibility
Were experiments performed using adequate number of biological replicates?
If 'No', please indicate reasons in Comments for Author box below.
Reviewer #1:
- Yes

Reviewer #2:
- Yes

Is there sufficient raw data to assess the rigor of the analysis?
If 'No', please indicate reasons in Comments for Author box below.
Reviewer #1:
- Yes

Reviewer #2:
- Yes

Does the methods section provide sufficient detail to permit reproducibility?
If 'No', please indicate reasons in Comments for Author box below.
Reviewer #1:
- Yes

Reviewer #2:
- Yes

Completeness

© 2026. Published by The Company of Biologists under the terms of the Creative Commons Attribution License (https://creativecommons.org/licenses/by/4.0/).

Are the manuscript's conclusions supported by the data?
If 'No', please indicate reasons in Comments for Author box below.
Reviewer #1:
- Yes

Reviewer #2:
- Yes

Scholarship
Do the authors cite and discuss the merits of data that would argue for and against their conclusion?
If 'No', please indicate reasons in Comments for Author box below.
Reviewer #1:
- Yes

Reviewer #2:
- Yes

Does the manuscript title & abstract accurately reflect the contents of the manuscript, without hyperbole?
If 'No', please indicate reasons in Comments for Author box below.
Reviewer #1:
- Yes

Reviewer #2:
- Yes

First revision

Author response to reviewers' comments

Response to reviewers – bio.062878

Every point below describes a change we made in the revised manuscript.

*Page and line numbers refer to the **highlighted** version of the revised manuscript, which carries continuous line numbering; the clean version is paginated identically. Where a change is diffuse rather than localised, we say "throughout".*

We thank both reviewers and the handling editor for their careful and generous reading. The reports were constructive and addressing them has helped to make the paper clearer and, in two places, more accurate. Below we respond to every point in turn. Reviewer comments are in italics; our responses follow. Changes are marked in blue in the highlighted version of the manuscript.

Editor

> *Please provide a point-by-point response… Please attend to all of the reviewers' comments.*

Done – this document.

> *Note that reviewer #2, minor issues #1: how you address this comment I leave to your discretion.*

We have taken the suggestion. The former Figures 3 and 4 are now a single two-panel Figure 3

© 2026. Published by The Company of Biologists under the terms of the Creative Commons Attribution License (https://creativecommons.org/licenses/by/4.0/).

(A, log odds ratio; B, Hedges' *g*), and the former Figures 6 and 7 are now a single two-panel Figure 6. This reduces the manuscript from seven figures to six and creates room for the new Figure 5 requested by Reviewer 2 (major issue 4), keeping us within the 8-display item limit. (Legends for the merged Figures 3 and 6 are in the Figure legends section, which begins on p20, L750.)

> *Please ensure your manuscript complies with our formatting guidelines.*

The manuscript has been reformatted throughout to incorporate the Company of Biologists LaTeX class, Biology Open section order (Introduction, Results, Discussion, Materials and Methods), 1.5 line spacing with continuous line numbering, Harvard author- date citations with an alphabetical reference list, an abstract of 196 words (p1, L11–21), a 29-word summary statement (p1, L24–25), a running title (p1, L26), the equal-contribution and co-senior-author note (p1, L27–28), figure legends collected at the end of the main text file rather than with the artwork (from p20, L750), and seven display items in total (six figures and one table).

Supplementary material has been revised to contain only data items. We moved the supplementary Methods and Results sections into the main article: Materials and Methods (p14–15, L527–567) and Results (p4–5, L147–172), and we now present the search strings as Supplementary Table S1 (cited at p9, L333).

Reviewer 1

> *This is an extremely comprehensive and impressive study…*

We are grateful for this compliment.

General feedback

> **1.** *Jones and DuVal studied female mate-choice copying only… the simplest fix would be to provide a female-only sensitivity analysis for the odds-ratio dataset.*

We have taken the first of the reviewer's two options. We refitted the combined odds ratio model restricted to data on female observers and report the results alongside the all- observer (male and female) estimate wherever we compare with Jones and DuVal's OR of 2.71.

The female-only mean OR is 1.81 (95% CI: 1.32 to 2.49; k = 164 effect sizes from 66 studies), against 1.81 (95% CI: 1.34 to 2.45) for the all-observer model (p3, L96–100). Restricting to female observers drops 8 male-observer effect sizes, all from our own extraction, and removes 3 studies entirely.

The two estimates are effectively identical, which we think is the more informative outcome: the gap between our pooled estimate and the reviewer's point of comparison, the OR of 2.71 in Jones and DuVal (2019), is not an artefact of having included male observers. It reflects the added evidence and the different analytical framework.

We also took the reviewer's comment as a prompt to fit observer sex as a moderator on its own. This moderator harmonizes cleanly with the Choosing_sex variable of Davies et al. (2020), so we fitted it in the new extraction on both scales and on the combined Hedges' *g* data. Male observers copied mate choices at least as strongly as females in every model fitted and no contrast was statistically distinct (new extraction: $F_{1,67}$ = 1.95, p = 0.17 on both scales; combined Hedges' *g*: $F_{1,225}$ = 0.17, p = 0.68). Because the male level is based on only 8 effect sizes from 5 studies, we report this as no evidence of a sex difference rather than as evidence of equivalence. Observer sex is now shown in one of the four moderator panels in the new Figure 5. The result is reported in Results at p4, L140–144, and the moderator itself is described in Materials and Methods at p14, L488–495.

> **2.** *The manuscript states that the project tested whether the 'main findings' of the two previous meta-analyses still hold, but it does not actually retest several of those findings.*

© 2026. Published by The Company of Biologists under the terms of the Creative Commons Attribution License (https://creativecommons.org/licenses/by/4.0/).

Agreed, and this was an overstatement on our part. Q1 now reads “Does the overall average effect reported by the two previous meta-analyses remain supported?”, and we state explicitly in the Introduction that Q1 concerns the pooled average effect rather than each individual moderator result. We adjusted the Abstract and the opening of the Discussion accordingly. The rescoped Q1 is at p2, L63–65; the corresponding Abstract sentence at p1, L13.

3. *The PRISMA diagram reports 40 + 58 + 29 = 127 total studies, but the two original datasets overlap. Doesn’t this mean that 127 is not actually the number of unique primary studies?*

The reviewer is right, and this is the same issue Reviewer 2 raises in major issue 2. We modified the PRISMA flowchart by adding an explicit node for studies shared between the two source datasets, and the Results now state the number of unique primary studies and explain why the three counts do not sum.

The corpus contains 94 unique primary studies, not the 127 the three source counts suggest. The 33-study difference has three parts:

- **30 studies are in both source datasets.** This is the correction the reviewer identified.
- **2 of our 29 new studies were already in Davies et al. (2020)** — Moran et al. (2013) and Ophir and Galef (2003). Both are pre-2019 papers that our multilingual search recovered independently and that we re-extracted from the primary reports; each is now counted once.
- **1 of the 40 study codes in Jones and DuVal (2019) duplicates another code in the same dataset** (GIE2018USI repeats GIE2018MAT: the same two reversal-versus- control contrasts appear under both codes).

There is no overlap between our extraction and Jones and DuVal (2019), and no study appears in all three sources. This passage is at p3, L83–91.

Working this out also corrected a claim in the submitted manuscript, which we are grateful to have caught. We had written that 13 of our 29 new studies fell within the original search windows “yet had not been included in them”. Two of those 13 — the Moran et al. (2013) and Ophir and Galef (2003) papers above — were in Davies et al. (2020); we had re- extracted them independently without recognising them as already-included studies. The correct figure is 11, and the Results now say so (p3, L73–75). The point the sentence was making still stands, and we have kept it: it is substantial to recover 11 pre-2019 studies that neither original meta-analysis included.

Because neither source dataset shares an identifier with the other, we resolved this by generating match candidates (same first-author surname prefix and publication year) and then adjudicating every candidate by hand against the source records and, where the two source teams disagreed, against the primary PDFs. The accepted and rejected candidates are both listed with reasons in the Supplementary Information. The full study-level crosscheck process is included in our archived repository.

Line-by-line comments and responses

All twelve line-by-line comments have been incorporated into the manuscript.

Comment (original submission)	Action	Now at
p2, L8-9 “moderately and positively biases”	Now “has a moderate positive effect on mate choice”	p1, L12

© 2026. Published by The Company of Biologists under the terms of the Creative Commons Attribution License (https://creativecommons.org/licenses/by/4.0/).

p2, L13 mixed UK/US spelling	Unified to UK spelling throughout	throughout
p4, L75 "in Open Science Framework"	Now "on the Open Science Framework"	p9, L290
p5, L90 "rather than entirely new synthesis"	Now "rather than an entirely new synthesis"	p9, L302
p6, L136–138 Google Scholar sentence	Rephrased as the reviewer suggested	p10, L338
p7, L149-150 "different target individuals that maintained a certain trait"	Definition of generalised *vs.* individual copying rewritten	p10, L356
p11, L245–246 which phylogeny is in which figure	Now states Fig. 2 shows the new extraction (15 species) and Supplementary Fig. S1 the combined dataset (33 species)	p13, L451
p12, L253 $r = 0.5$ *vs.* $p = 0.5$	ρ used consistently throughout	p13, L458 (first use); throughout
p18, L427 duplicate $p = 0.72$	Duplicate removed	p4, L140
p20, L493–495 inconsistency with the Methods	Reconciled — see below	p6, L215
p22, L544 "The honest interpretation is…"	Now "A cautious interpretation is…"	p7, L253
p22, L550 comma after "ways"	Added comma	p7, L258

On p20, L493-495 the reviewer correctly spotted a contradiction. We extracted experimental design and choice outcome, as the Methods state. The Discussion now distinguishes the two cases: those two variables were extracted but are confounded with species and assay tradition in the combined corpus and were not pre-registered as moderators, so we did not fit them; live versus video demonstrators and the presence of a control group were not coded at all.

Reviewer 2

> *I believe it is an important work… the current version of the article is already very close to being suitable for publication.*

We thank the reviewer for this assessment and for the specific suggestions.

Major issues

> **1.** *The preregistration protocol could not be retrieved.*

We are sorry the reviewer had trouble accessing it. We rechecked the OSF project (https://osf.io/vfahk/overview), and the protocol PDF is publicly accessible. To remove any dependence on OSF availability, we also deposited the protocol in the GitHub repository and included it in the Zenodo archive, and the Data and resource availability statement now reflects this (p16,

© 2026. Published by The Company of Biologists under the terms of the Creative Commons Attribution License (https://creativecommons.org/licenses/by/4.0/).

L597–599).

> **2.** *It is unclear which databases were used… how many records were obtained from each… how the 898 records were excluded… and how the study counts reconcile.*

We made four changes in response to this comment:

- The Methods now names all five sources and states that no others were used: Web of Science Core Collection, Scopus, Google Scholar, SciELO, and BASE. The phrase "including X, Y, Z" has been removed. See p9, L330–333.
- Supplementary Table S1 gives, for each source, the full query string, the access date and the number of records retrieved (2,855 in total). The identification box of the revised PRISMA flowchart now carries the same eleven per-source counts, which sum to the 2,855 total. Supplementary Table S1 is cited at p9, L333.
- The PRISMA flowchart now shows explicitly how the 898 pre-2019 records inherited from the two source meta-analyses were handled: **our study inherited their exclusion from the original reviews.** These records were already screened and excluded by the two previous teams within their own search windows, and we did not re-adjudicate those decisions. The corresponding box is labelled "Pre-2019 records already screened by the two previous reviews; exclusion inherited, not re- screened", so a reader can see the provenance of the 1,056 records that entered our own screening rather than having to infer it. The accompanying Methods passage is at p10, L345–351. We note the implication in the Methods.
- The number of studies shared between the two source datasets is now stated in the flowchart's "Previous studies" box, the final box reports the 94 unique primary studies alongside the 33 duplicate study records removed, and the Results explain why 40 + 58 + 29 is not a count of unique studies.
- A further point of transparency the reviewer did not ask for: many retrieved records, particularly the non-English ones, carried truncated abstracts, which limits what a title-and-abstract screen can see. We recovered the full abstracts with the ris-enrichtool (Santos, 2026; SciELO Preprints, doi:10.1590/scielopreprints.15571) before screening, and the Methods now state this (p10, L339).
- One further change the reviewer did not ask for but which follows from this: the flowchart is now generated from a single table of counts by the PRISMA2020R package rather than drawn in the PRISMA2020 Shiny app, so the figure and its numbers are version-controlled and reproducible from the archived repository. The Methods have been corrected accordingly.

On the reviewer's last point: the totals from the Discussion (227 Hedges' *g* from 87 studies; 172 log odds ratios from 69 studies; 33 species) now also appear in Results, under *Screening outcomes and dataset characteristics* (p3, L83–85).

> **3.** *Was the taxonomic-group analysis performed by Jones & DuVal and/or Davies et al., or is it original?*

Davies et al. (2020) fitted taxonomic group, and it was one of only two of their ten moderators to reach significance ($Q_M = 8.57$, $P = 0.04$), with copying strongest in mammals, followed by birds and fish, and weakest in arthropods. The Results and Discussion now state this and draw the comparison. On the Hedges' *g* scale our combined analysis recovers the same ordering, but the contrast is no longer statistically supported once the new evidence is added ($F_{2,27} = 3.00$, $p = 0.066$), and on the log odds ratio scale the ordering does not hold. We also note that their mammal estimate — the level that drove the gradient — rested on six effect sizes from two species. We therefore frame this analysis as revisiting an existing result on an expanded dataset rather than as a new question, with the substantive finding being that the taxonomic contrast weakens and becomes scale-dependent. The Results passage is at p4, L126–132.

> **4.** *A few biological moderators are discussed extensively but their numerical results are only described in the text. I would recommend a figure or table.*

Added as new Figure 5: orchard plots of every biological-moderator level on both scales. Merging the

© 2026. Published by The Company of Biologists under the terms of the Creative Commons Attribution License (https://creativecommons.org/licenses/by/4.0/).

figures per your minor issue 1 created the room for it within the eight-item limit.

The new figure is a two-column grid — Hedges' *g* on the left, log odds ratio on the right — with one row per moderator: observer sex, observer mating status, copying mechanism and social-cue modality. Each cell shows the marginalised mean, 95% confidence and prediction intervals, and *k* (with the number of studies) for every level. Because the four moderators harmonise with the source datasets to different extents, each cell uses the widest dataset available for that moderator-and-scale combination and states which one in its sub-panel heading, so it is visible at a glance which estimates rest on the combined corpus and which on the new extraction alone.

Note that observer sex is a new fifth moderator added at revision, prompted by Reviewer 1's general comment 1; the submitted version fitted four. The Results sentence pointing to Figure 5 is at p4, L145–146.

Minor issues

> **1.** *Consider whether some figures could be combined into single multi-panel figures (e.g., Figures 3 and 4, or Figures 6 and 7).*

Done, exactly as suggested — see the response to the editor above.

> **2.** *The findings from small-study and publication-bias assessments, time-lag patterns and sensitivity analyses could be better described in the Results and further explored in the Discussion.*

Agreed, and this also aligns with the journal's rule on supplementary text files. These findings now appear in full in the Results, with all numerical values (Egger's slopes, both corrected means on both scales, leave-one-out and leave-one-species-out ranges, and the contentious-effect-size sensitivity analysis), and are interpreted in the Discussion. The supplementary now holds only the supporting funnel, leave-one-out and sensitivity figures. In the revised manuscript these Results paragraphs are at p4–5, L147–172 (small-study effects and time-lag at L148–153, the two corrected means at L154–159, leave-one-out at L160–167, and the contentious-effect-size sensitivity analysis at L168–172); the corresponding Methods subsections are at p14–15, L527–567, and the interpretation is drawn together at p8, L282–283.

Second decision letter

MS ID#: bio.062878R1

MS Title: Mate choice copying in non-human animals: an update of two meta-analyses

Authors: Eduardo S. A. Santos; Aleksandra Milenović; Aneta Arct; Anna Lenz; Ayumi Mizuno; Cassidy Schneider; Christine Sosiak; Erick Lundgren; Hao Qin; Iwo Gross; Jimuel Celeste; Kyle Morrison; Mahi Zakir; Marija Purgar; Santiago Ortega; Sergio Hernandez; Shinichi Nakagawa; Malgorzata Lagisz

Thank you for submitting a revised manuscript and addressing the reviewer comments. After reading through your revisions and discussing them with the reviewers, I am happy to tell you that your manuscript has been accepted for publication in Biology Open, pending our standard publication integrity checks. It was accepted on 17th September 2026.

© 2026. Published by The Company of Biologists under the terms of the Creative Commons Attribution License (https://creativecommons.org/licenses/by/4.0/).
